# Volatile Aroma Compounds of Brandy ‘Lozovača′ Produced from Muscat Table Grapevine Cultivars (*Vitis vinifera* L.)

**DOI:** 10.3390/molecules24132485

**Published:** 2019-07-06

**Authors:** Saša Matijašević, Jelena Popović-Djordjević, Renata Ristić, Dušica Ćirković, Bratislav Ćirković, Tatjana Popović

**Affiliations:** 1 Department of Horticulture, Faculty of Agriculture, University of Belgrade, Nemanjina 6, 11080 Belgrade, Serbia; 2 Department of Food Technology and Biochemistry, Faculty of Agriculture, University of Belgrade, Nemanjina 6, 11080 Belgrade, Serbia; 3 ARC Training Centre for Innovative Wine Production, School of Agriculture, Food and Wine, University of Adelaide, PMB 1, Glen Osmond, SA 5064, Australia; 4 College of Agriculture and Food Technology, Ćirila i Metodija 1, 18400 Prokuplje, Serbia; 5 Faculty of Agriculture, University of Priština, Kopaonička bb, 38219 Lešak, Serbia; 6 Faculty of Biotechnology, University of Montenegro, Mihaila Lalića 1, 81000 Podgorica, Montenegro

**Keywords:** grape brandy ‘Lozovača′, Radmilovac Muscat, Banat Muscat, pectolytic enzymes, terpenes, esters, PCA, AHC, GC/MS.

## Abstract

Grape brandy, known as ‘Lozovača’, is one of the most produced alcoholic beverages in the Republic of Serbia. Muscat cultivars are highly priced in grape brandy manufacturing. Among the numerous factors, cultivar-specific characteristics have a significant influence on its quality and aroma profile. Pectolytic enzymes play a part in increasing intensity of the prefermentative aroma by hydrolysis of terpenic glycosides, from which the compounds that contribute to the aroma of brandy are released. In this study, grape brandy samples were produced from five Muscat table grapevine cultivars (*Vitis vinifera* L.) namely, Early Muscat, Radmilovac Muscat, Banat Muscat, Italia Muscat, and Muscat Hamburg, with the addition of pectolytic enzyme in two different concentrations or without it (control). A total of 58 volatile aroma compounds were detected by means of combined gas chromatographic-mass spectrometric (GC/MS) method. Ethyl esters of C_8_–C_18_ fatty acids (21) and terpene (16) compounds were considerably more abundant in all grape brandy samples compared to the other volatile compounds identified. Pectolytic enzyme, positively affected terpenes content in the brandy of all studied cultivars. The similarities between brandy samples produced from Muscat Hamburg (MH) and other Muscat cultivars may be attributed to the parentage of MH to those cultivars.

## 1. Introduction

Brandy is a popular spirit which is produced in a few regions worldwide and it falls into the fifth biggest category of spirit drinks (1.2 billion liters of brandy out of 20.0 billion liters in total). European Union legislation describes brandy as an alcoholic beverage which is produced from wine spirit, with or without wine distillate, distilled at less than 94.8% (*v/v*), provided that the distillate is not greater than the maximum of 50% of alcoholic content in the final product [1].

Aromatic compounds found in grape berries are most commonly found in the epicarp layer of the skin and in smaller quantities in the pulp. As free volatile compounds, they contribute directly to the aromatic profile of beverages, whereas when linked to sugars in the form of glycosides, such as β-d-glycosides; α-l-rhamnopyranosyl-β-d-glycosides; α-l-arabinofuranosyl-β-d-glycosides and β-d-apo-furanosyl-β-d-glycosides, these compounds have no smell [1,2]. The release of bound volatile compounds can be achieved by physical (temperature), chemical agents (acidification), and biochemical methods (enzymes) which are the most effective. Enzymes break the glycosidic bond without disturbing the aromatic profile which occurs during acid hydrolysis. The application of enzymes in the oenological sector dates from the late 1970s [3]. The increase in the intensity of the prefermentative aroma is attributed to the effect of the pectolytic enzyme on the pomace, and its contribution to the hydrolysis of terpenic glycosides from which the compounds contributing to the aromas are released [4,5]. Certain aromatic precursors that are present in the must cannot be transformed without the presence of the appropriate yeast set of enzymes.

The grape aroma is synthesized in grape berries by a variety of enzymes. Genetic variation in aroma biosynthesis genes causes differences in aroma between grapevine cultivars: an allelic variant of 1-deoxy-d-xylulose-5 phosphate synthase, a terpenoid biosynthetic gene, causes accumulation of terpenoids in Muscat grapes [6,7,8]. The genetic factors underlying the aroma typicity of grapevine cultivars remain unexplored, even though in Europe alone over 2000 cultivars have been described [9]. Glycosides are not volatile, so they do not directly contribute to wine aroma. However, they affect the aroma indirectly as they form a precursor pool from which volatile aglycones can be released during yeast and malolactic fermentation, during vinification by adding exogenous glycosidases, during wine aging owing to its low pH, and, as demonstrated recently, by enzymatic hydrolysis in the mouth, catalyzed by the enzymes in the saliva [10,11]. 

Grape brandy quality is dependent on a number of factors, most notably cultivar-specific characteristics, grape processing method, alcoholic fermentation, and distillation method [12]. Apart from water and ethanol, as the main constituents, grape brandy also contains a number of other components whose concentration mostly depends on the cultivar i.e., raw materials used and the technology employed (fermentation method, distillation process, etc.) [13]. The aroma of a grape product is the result of simultaneous activities of many aromatic substances. Some grape products require the presence of a few compounds that give them their cultivar-typical aroma, whereas some others have their distinctive character generated by only a wide range of aromatic substances occurring at particular ratios [1]. Higher alcohols are quantitatively the largest group of volatile compounds found in distillates, giving them their distinctive aroma, flavor and fundamental character [13,14]. 

The aromatic potential of different grape cultivars is of special importance for grape brandy quality. The most important factors affecting the aroma in Muscat and non-Muscat cultivars are terpenic and aliphatic alcohols, respectively [15]. Regarding Muscat cultivars, this potential arises from terpenic content [1,3]. One of the world’s most famous beverages which is similar but not identical to grape brandy, the so-called Pisco is produced in some countries of South America (Chile, Peru and Argentina) by distillation of, mostly, Muscat cultivar wine, while in Italy, it is marketed under the name Acquavite d’uva. 

Muscat Hamburg is an economically very important table grape cultivar and is often highly priced due to its enjoyable taste [16]. ‘Lozovača’ is a famous grape brandy in Serbia, obtained through fermentation and distillation of the whole non-strained mash of grapes. ‘Lozovača’ produced from noble Muscat Hamburg grape (*Vitis vinifera* L.) is especially valued because of the specific aroma. Owing to the richness of aromatic compounds in Muscat cultivars, they are often appraised as models for the study of flavor compounds [4,17]. However, our previous work demonstrated significant effects of evaluated cultivars, Demir Kapija, Early Muscat, Radmilovac Muscat, Banat Muscat, Black Muscat, Smederevo Muscat, Italia, and Dattier on the aroma profiles of produced brandies [17].

The objective of this study was to evaluate the composition of the aromatic complex of the grape brandy ‘Lozovača’ produced with the application of pectolytic enzyme from five Muscat table grapevine cultivars. Domestic cultivars, Radmilovac Muscat and Banat Muscat, and the introduced cultivars, Early Muscat, Italia Muscat, and Muscat Hamburg, were used for the brandy production. The effect of the enzyme on the release of the aromatic compounds, as well as the similarities between the composition of brandy samples produced from Muscat Hamburg and other Muscat cultivars were discussed. 

## 2. Results

### 2.1. Volatile Compounds Composition of Brandy Samples 

In the individual samples of Early Muscat (EM), Radmilovac Muscat (RM), Banat Muscat (BM), Italia Muscat (IM) and Muscat Hamburg (MH) a total of 37, 35, 25, 32, and 27 aromatic compounds, respectively were identified that belong to different groups including acetals, alcohols, acids, esters, terpenes, ketones, and amides. The major aroma contributing compounds alcohols, acids, esters, terpenes are presented in Table 1.

Alcohols—All brandy samples (EM, RM, BM, MH, and IT) were found to contain 1-hexanol and phenyl–ethyl alcohol. Phenyl–ethyl alcohol was identified in all distillate samples except for the V2 variant in the brandy of the Radmilovac Muscat cultivar. The smallest percentage was recorded in the control sample of the Radmilovac Muscat distillate (0.42%) and the highest relative representation was recorded in the Muscat Hamburg V1 distillate (25.43%). The smallest content of phenyl–ethyl alcohol was recorded in the brandy of Italia Muscat cultivar (C—5.6%, V1—1.54% and V2—3.51%) in comparison to other distillates. It is important to note that with the use of pectolytic enzymes in the Italia Muscat brandy, the relative content of phenyl–ethyl alcohol decreased. Notably higher relative content of 1-hexanol in relation to other cultivars were found in distillates from Banat Muscat (C—8.18%, V1—6.74%, V2—3.41%), Radmilovac Muscat (C—6.01%, V1—1.9%, V2—9.12%) and Muscat Hamburg (C—3.61%, V2—6.17%), Table 1.

*Acids*—Short-chain fatty acids have not been identified in any sample (either in the control sample or in the variants with the implementation of pectolytic enzymes). Of the middle-chain fatty acids, octanoic, decanoic, dodecanoic and hexadecanoic acids were identified in most brandy samples (Table 1). These volatile acids were identified sporadically in the investigated distillates. Octanoic acid was present in all brandy samples obtained from the Early Muscat cultivar (relative content of C—0.32%, V1—0.63% to V2—0.88%). In brandies of other cultivars, this acid appeared sporadically in the range of 0.1% to 5.0%. Decanoic acid, similarly to the previous one, was present in all samples of the Early Muscat distillate (C—1.25%, V1—4.91% and V2—6.01%). It should be noted that the relative content of decanoic acid was slightly higher than the octanoic, and especially in relation to the hexanoic acid, which was only sporadically identified. Dodecanoic acid had the highest relative abundance as compared to the other fatty acids in all analyzed distillate samples; from 0.11% (MHC) to 6.61% (EMV2). From the obtained results, it could be noticed that the application of pectolytic enzyme in most cases influenced the increase in the relative content of volatile acids.

*Esters*—In relation to the content of the medium-long organic acid chain, the medium and long chain of ethyl esters was also present. Results of the aromatic compounds identified in this study show that ethyl esters of C_8_–C_18_ fatty acids were the most numerous and the most abundant in all samples (Table 1). The relative content of ethyl octanoate, ethyl decanoate, and ethyl hexanoate was higher in grape brandy produced from cvs. Muscat Hamburg, and Italia Muscat than in those from cvs. Early Muscat, Radmilovac Muscat and Banat Muscat; among them ethyl decanoate and ethyl octanoate were the most abundant. In ethyl esters of long-chain fatty acids, the relative content of ethyl dodecanoate, ethyl hexadecanoate, ethyl linoleate and ethyl tetradecanoate were the highest. In addition, the samples had a significant relative content of ethyl 9-hexadecanoate and ethyl stearate. The predominant esters were ethyl hexadecanoate and ethyl linoleate.

*Terpenes*—The total relative content of terpenes varied (Table 1), in the control sample from 4.80% (Banat Muscat) to 24.78% (Radmilovac Muscat), in the sample with a lower dose of enzyme (0.3 g/kg) from 8.05% (Italia Muscat) up to 50.85% (Early Muscat) and in the sample with the higher dose of enzyme (0.7 g/kg) from 11.07% (Italia Muscat) to 34.78% (Radmilovac Muscat).

**Figure 1:**
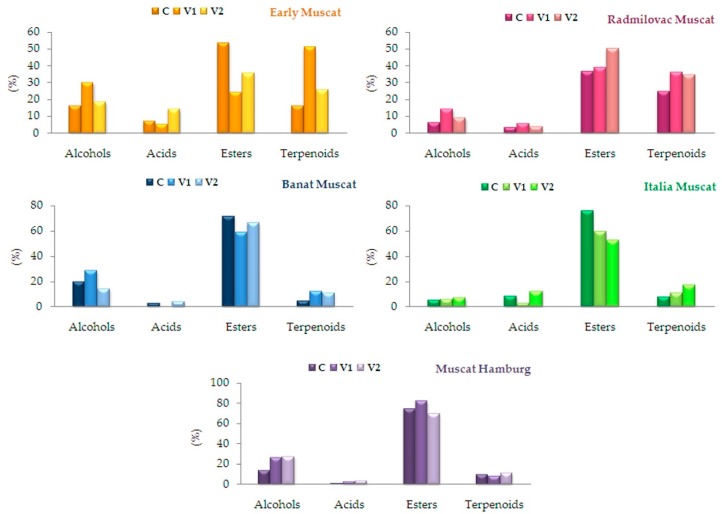
Changes of relative abundance of aromatic compound total content in grape brandies in different experiments; C—control (no enzyme), V1 and V2—0.3 g and 0.7 g of pectolytic enzyme per kg of grape, respectively.

The similarity regarding the relative abundance of total esters (70%) was observed between the samples of Muscat Hamburg, Banat Muscat and Italia Muscat. On the other hand, the effect of pectolytic enzyme on the relative abundance of terpenes in Early Muscat, Ramilovac Muscat and Banat Muscat samples had a similar pattern. Namely, in V1 samples (with the lower amount of enzyme applied) the higher relative content of terpenes was observed compared to the control sample and V2 samples, Figure 1. 

### 2.2. Principal Component Analysis and Hierarchical Component Analysis

**Figure 2:**
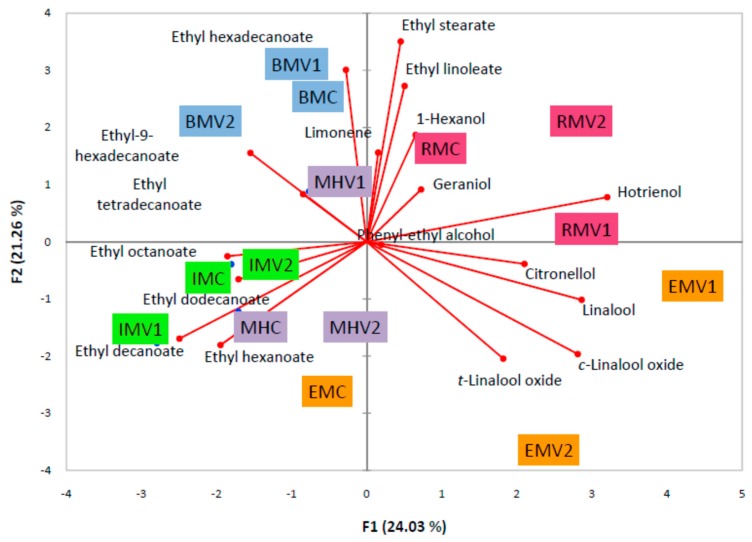
Principal component analysis biplot of the volatile aroma compounds identified in grape brandy samples of Muscat cultivars.

The most abundant volatile aromatic compounds were used in PCA analysis (marked with * in Table 1). Principal component analysis) revealed separation of evaluated samples based on 6 components with eigenvalue >1. The first PC explaining 24.03% of variance was mostly determined by terpenes, citronellol, hotrienol, linalool, *c*-linalool oxide and, esters, ethyl hexanoate, ethyl decanoate, and ethyl dodecanoate (Figure 2). Ethyl esters hexadecanoate, linoleate and stearate, and terpenoid *t*-linalool oxide provided loadings on PC2 that explained further 21.26% of variance. The third PC explained 13.83% of variance with loadings of 1-hexanol, ethyl octanoate, ethyl tetradecanoate and ethyl-9-hexadecanoate, while the fourth PC explained 10.99% due to the loading of phenyl–ethyl alcohol and limonene. 

Samples of RM were positioned relatively close in the top right side based on their high relative content of geraniol, 1-hexanol (RMC) and hotrienol (RMV1). Close to RMV1 was EMV1, characterized by citronellol and linalool, while EMV2, in the bottom of the right quadrant, was separated from their counterparts by linalool oxides, indicating significant effects of different concentrations of used pectolytic enzyme on aroma profiles of EM brandies. A similar effect was observed for Muscat Hamburg; MHC and MHV2 were positioned in the bottom left quadrant due to high content of ethyl hexanoate, while MHV1 was more associated with higher content of limonene and ethyl tetradecanoate. The group of IM samples were positioned closely in the left bottom quadrant and were characterized by higher content of ethyl octanoate, decanoate and dodecanoate. Similarly, the group of BM samples located in the top right quadrant was separated by ethyl hexadecanoate (BMV1) and ethyl-9-hexadecanoate (BMV2). 

To explore the influence of used pectolytic enzyme on aroma profiles in more detail, principal component analysis was performed for each variety (Supplementary Materials Figure S1). While 2 PCs explained 100% of variance for all treatments, the loadings of components on the first or second PC varied. For MH and RM almost all alcohols and esters were loaded on PC1 (except ethyl decanoate for MH and phenyl–ethyl alcohol for RM). For RM also all terpenes (except geraniol) characterized PC2 which explained further 40% of total variance. Other varieties, BM, IM, and EM, were separated by different compounds. However, there was a clear separation of treatments with lower or higher application of enzymes. For BM the highest loadings on PC1 were provided by ethyl octanoate and decanoate, and ethyl hexanoate, ethyl-9-hexadecanoate and linalool on PC2. IM was characterized mostly by 1-hexanol, ethyl tetradecanoate, limonene, *c*-linalool oxide and hotrienol on PC1, and ethyl decanoate, ethyl-9-hexadecanoate and stearate on PC2. Separation of EM samples was mostly based on ethyl octanoate, dodecanoate, stearate and terpenoids, limonene, linalool, hotrienol and citronellol (PC1) and phenyl–ethyl alcohol, ethyl-9-hexadecanoate and *t*-linalool oxide (PC2). 

**Figure 3:**
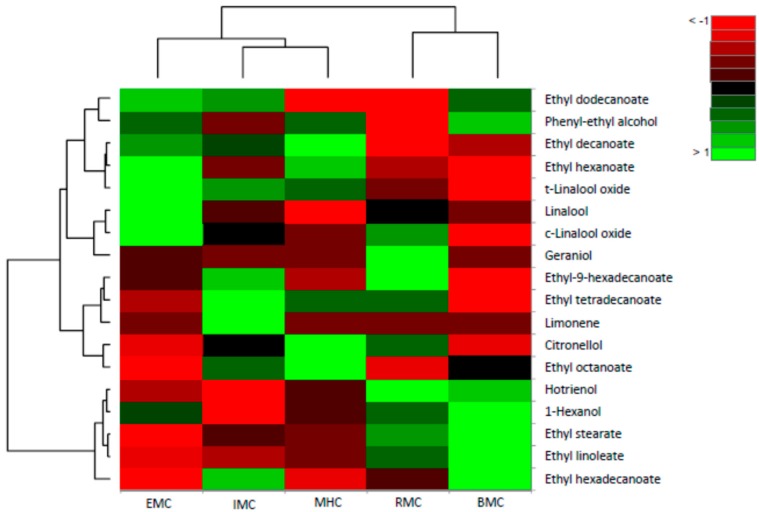
Heatmap of the volatile aroma compounds identified in grape brandy samples of Muscat cultivars without the addition of enzymes (control) based on relative abundance differences of compounds.

The agglomerative hierarchical clustering (AHC) of brandy samples without the addition of pectolytic enzyme grouped samples by their dissimilarity into three clusters; a cluster (C) with EMC, IMC and HMC, and two clusters that contained only RMC or BMC (Figure 3). The biggest dissimilarities between 5 varieties were found in the content of phenyl–ethyl alcohol, ethyl decanoate, hexadecanoate, dodecanoate, octanoate, linoleate, and to a lesser extent, linalool and geraniol. The biggest similarity was observed between MHC and IMC, according to the content of ethyl linoleate, 1-hexanol and hotrienol, linalool, linalool oxide-t, ethyl tetradecanoate and ethyl octanoate (Supplementary Materials Figure S2).

**Figure 4:**
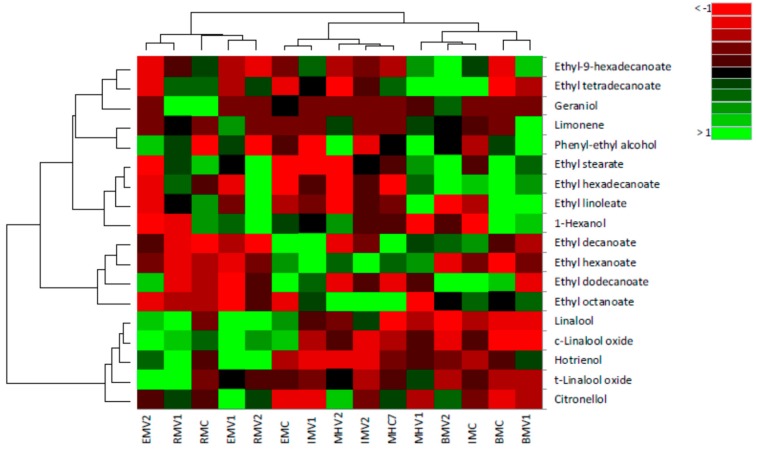
Heatmap of the volatile aroma compounds identified in grape brandy samples of Muscat cultivars with and without the addition of enzymes (control) based on relative abundance differences of compounds.

The agglomerative hierarchical clustering (AHC) of brandy samples with and without the addition of pectolytic enzyme grouped samples into three clusters. The C1 included EMC, IMV1, IMV2, MHC, and MHV2, the second cluster (C2) contained EMV1, EMV2, RMC, RMV1, and RMV2 while the third cluster (C3) had BMC, BMV1, BMV2, IMC, and MHV1 (Figure 4). 

C1 was characterized by high content of ethyl esters: hexanoate, octanoate, decanoate, and low level of ethyl hexadecanoate and linoleate compared to the other two clusters. C2 was found to be quite opposite, the same esters were quite low but all terpenes high. C3 had a high content of phenyl–ethyl alcohol, ethyl hexadecanoate and linoleate, but low levels of terpenes: linalool, citronellol, and geraniol (Supplementary Materials Figure S3).

## 3. Discussion

Being a product of wine distillation, brandy does not contain the non-distillable wine organic and inorganic compounds. Non-volatile organic acids are not present in the distillate which affects the taste balance and the high content of alcohol in brandy (about 40% *v/v*) that causes a burning sensation in the mouth and increases the sweetness. Grape brandies have a composite aroma profile with hundreds of volatile compounds found in grapes (primary aromas) and/or during wine fermentation (fermentation aromas), whereas some arise from the distillation process or through extraction from oak wood. Many significant aroma compounds are formed during grape ripening (monoterpenes) and during alcoholic fermentation (higher alcohols, middle chain mono-carboxylic acids and mono-carboxylic acids). The organoleptic characteristics could be distinctive even though the volatile composition is fairly similar, and this is possibly because of slightly different concentrations of volatile compounds [1]. 

*Alcohol*—During the production of wine, C6 alcohols are formed mainly during prefermentative production steps (harvesting, transport, crushing, mashing, and pressing of the grapes), by enzymatic degradation and subsequent reduction of long-chain fatty acids [18,19]. A significant amount of C6 alcohols from grape skins is extracted to the must during fermentative maceration [20]. According to the literature, 1-hexanol is denominated as a rough indicator of the pressing degree [21]. In this respect relative content range in the studied brandy samples (C, V1, and V2) made from the same cultivar imply a possible influence of the pressing process. The application of the pectolytic enzyme leads to the increase of the relative abundance of phenyl–ethyl alcohol in brandy samples of most cultivars, compared to control ones, except for the samples produced from IM and RM. In case of 1-hexanol, the enzyme applications affected its relative content in samples EMV1, RMV2, IMV1, IMV2, and MHV2 (Table 1) with possible effects on their aroma profiles. In brandy samples the most abundant alcohols are associated with flower, green, cut grass, grass and herbaceous aromas (1-hexanol), floral, rose, and honey aromas (phenyl–ethyl alcohol), while 1-heptanol is associated with oily aroma (Table S1, Supplementary Material). 

*Acids*—Short-chain fatty acids were not identified in any sample, either in the control sample or in any other variant with the application of pectolytic enzyme (Table 1). These compounds are usually considered as negative aroma contributors, with sharp smells described as rancid, fatty, and cheesy [13,22]. Middle-chain fatty acids usually do not exhibit important effects on the aroma of distillates due to relatively high odor perception thresholds [13,23,24]. Moreover, the impact of aromas that these acids produce is described as restrained, but unpleasant [22,25]. It has been previously shown that fatty acids such as octanoic, decanoic and hexadecanoic acids mostly impart unpleasant odors of rancid fat, greasy oils, lard or spoiled cheese [22,26,27,28]. Octanoic acid was detected in samples EMC, EMV1, EMV2, BMV1, BMV2, IMC, IMV1, MHC, and MHV2, whereas hexadecanoic acid was found in EMC, EMV2, RMC, RMV1, RMV2, and MHC samples (Table 1). 

*Esters*—Esters are formed during the alcoholic fermentation. Most esters are products of yeast metabolism or are formed by esterification of fatty acids in the presence of ethanol in high concentrations during ageing of the distillate [29]. Accordingly, esters represent the most common class of compounds that contribute to the aroma in the brandy [30]. The results obtained in this study are in line with literature [31]. 

The ethyl esters produced during raw material fermentation are transferred into the alcoholic beverage and their content may increase or decrease during wine ageing [2,26,27]. Fatty acid esters contribute to the pleasant fruity and floral aroma of the distillate [13,32]. Of special importance are ethyl esters of middle-chain fatty acids due to high concentrations in alcoholic beverages and their volatility, as they positively contribute to the aroma with odors reminiscent of fruit (green apple, pear, and grapes) and/or soap [33]. For example, ethyl octanoate imparts a pleasant fresh fruity aroma [29], ethyl hexanoate produces a tropical fruit odor and aroma, whereas ethyl octanoate and ethyl dodecanoate give a pear-like aroma and a characteristic fruity aroma, respectively [28]. Ethyl esters are the most abundant chemical class of aroma factors in cognac. Furthermore, the importance of ethyl hexanoate is particularly highlighted in imparting sensory attributes of strawberries and anise [30]. Ethyl hexanoate, ethyl octanoate and ethyl decanoate are the most abundant in apple and apricot distillates [27]. In the current study the highest relative abundance (%) of ethyl hexanoate was found in the sample of Italia Muscat (IMV1). The influence of the applied pectolytic enzyme was noticed for ethyl octanoate in the following samples: RMV1 and RMV2, BMV1, IMV2, and MHV2. In these samples the relative abundance compared to the control sample was detected. The negative influence of the addition of pectolytic enzyme, the decrease in the relative abundance of ethyl dodecanoate, was noticed in brandy samples (V1 and V2) of Early Muscat and Italia Muscat (Table 1). Ethyl decanoate was present in the highest percentage in the samples of Italia Muscat (IMC and IMV1), Muscat Hamburg (MHC) and Early Muscat (EMC). Ethyl hexadecanoate and ethyl linoleate were present in all examined brandy samples, Table 1. The fruity sweet aroma suggestive of bananas and apples is related to ethyl butanoate; a vinous, apple, and banana-like aroma to ethyl hexanoate; a banana-, pineapple-, and brandy-like aroma to ethyl octanoate; a brandy, oily, fruity, and grape-like aroma to ethyl decanoate; lard and soap-like odor to both ethyl dodecanoate and ethyl tetradecanoate [27,29]. 

*Terpenes*—The main aromatic substances of grape (*V. vinifera*), overripe pomace and wines of Muscat cultivars are terpenes, linalool, geraniol and nerol, but also terpineol [20]. Muscat grape cultivars are particularly rich in terpenes [1]. Linalool, nerol, and geraniol, independently of each other, do not dominate in the muscat aroma of Muscat cultivars. However, in combination, they give a floral and fruity character that resembles the character of muscat aroma [34]. Terpenes in grapes, on one hand, are found in the free form and then they are aromatic, but on the other hand, they are found in the form of precursors of glycosides and polyols, which are non-aromatic. In grapes of Muscat cultivars, terpenic glycosides are present in about 77%, and free terpenes about 23%. Aromatic compounds bonded in the form of glycosides are more present than the free compounds (3 to 10 times) regardless of the cultivar [35]. The substances responsible for the specific aroma of Muscat cultivars are mainly found in the skin of the berries [3]. Geraniol and nerol are mainly present in the skin of the berry, while linalool is significantly evenly distributed between the juice and the firm parts of the berry [36,37]. The aromatic compounds found in trace amounts in grape brandies such as *α*-terpinolene, hotrienol, rose oxide, citral, citronellol, manoyl oxide, myrcene, *α*-terpinene, and *p*-cymene, significantly contribute to the grape brandy aroma and are specific only for distillates obtained from grapes (*Vitis vinifera* L.) [15,16]. 

A total of 16 terpene compounds were identified and observed in all samples of the studied cultivars (control and with the application of enzymes). Among them, linalool, citronellol, hotrienol, *c*-linalool oxide, *t*-linalool oxide, limonene, and geraniol were the most abundant, and among them, the most dominant were linalool and citronellol. Other terpene compounds were detected sporadically and in significantly less relative content (α-pinene, γ-terpinene, α-terpinolene, rose oxide, neroloxide, α-terpineol, farnesol, β-fenchene, and epoxylinalool). Linalool was detected in all samples (1.73%–19.22%), whereas nerol was not present at all. The results obtained for the linalool content indicated its increase in the samples with added pectolytic enzyme of all studied cultivars except for Banat Muscat (Table 1). Geraniol was the most abundant in Radmilovac Muscat control (6.8%), but interestingly it was not detected in any sample of Italia Muscat. Brandy sample of Early Muscat cultivar (EMV1) was the richest in citronellol (17.05%, Table 1). Hotrienol was detected in relatively high percentage (5.98%, 5.92%, and 7.17%) in samples EMV1, RMV1, and RMV2, respectively (Table 1). *c*-Linalool oxide was found in all samples (0.38%–3.21%), except for BMC and BMV1. *t*-Linalool oxide was not identified only in Banat Muscat brandy samples, while in other samples its relative content ranged from 0.23%–3.37%. Limonene, associated with lemon and fruity aromas, was detected in the samples where pectolytic enzyme was applied (EMV1, RMV1, BMV1, BMV2, MHV1, and MHV2), which may be attributed to the influence of the enzyme. The effect of compounds on the impartment of both odor and aroma is mostly induced by its abundance i.e., content. However, this is not the case with terpenic compounds and some esters which were noted for their low olfactive threshold values. Namely, the low detection threshold level indicates a high degree of contribution to the distillate aroma regardless of their low concentration. Linalool and geraniol, for example, with low detection threshold, have a far stronger aromatic character as compared to nerol that reaches identical odor intensity at four-fold concentrations [38]. Terpenes are mostly responsible for fine aromatic, flowery and floral aromas [39,40,41]. 

The most notable changes in relative abundance (%) were observed for esters and terpenes. The application of pectolytic enzyme influenced the increase of the relative content of esters in the samples of RM and MH. On the other hand, in brandy samples obtained from other studied cultivars, relative content of esters declined with the application of the pectolytic enzyme. Pectolytic enzyme, in both concentrations, positively affected terpene content in brandy samples of all studied cultivars. 

## 4. Materials and Methods

Experiment was carried out on the plantation of grapevine cultivars at the Radmilovac Experimental Field (Faculty of Agriculture, University of Belgrade, Belgrade, Republic of Serbia). The geographical position of the plantation is at 44° 45′ N/20° 35′ E and 135 m above sea level on a mild slope exposed to the south. By its location, the plantation belongs to Belgrade area and Grocka vineyards. Grocka vineyards are characterized by favorable climatic conditions (vicinity of the Danube River) for the production of high-quality table grapes. The examined cultivars were inoculated on the vine rootstock *Riparia × Berlandieri Kober 5BB*. Plant density is 3 m between rows and the distance between the vine-wood is 1 m. Plant population per hectare is 3333 vine-wood. For the production of the brandy, grapes of table cultivars Early Muscat (EM), Radmilovac Muscat (RM), Banat Muscat (BM), Italia Muscat (IM), and Muscat Hamburg (MH) were used. RM and BM are native cultivars bred in 1980s at the Faculty of Agriculture, whereas EM, MH and IM are introduced cultivars. Their description is given according to literature [42,43].

### 4.1. Cultivars Description

Early Muscat is a table cultivar of early stage of ripening (the end of August, OIV Code N_o_ 304 -3- early). It was created by crossing the cultivars Muscat Hamburg x Queen of the Vineyard. Its cluster is of medium size, nice looking. The berries are oval, medium-sized, yellow, and white. The flesh is tendinous with a fine Muscat taste.

Radmilovac Muscat is an early table cultivar (the beginning of September, OIV Code N_o_ 304 – 3 - early). The cultivars Queen of the Vineyard x Muscat Hamburg were used as parental partners. Its cluster is medium to large-sized, loose. The berries are large, round, the skin is yellow-green. The flesh is crispy with a pleasant Muscat taste.

Banat Muscat is an early table cultivar (the beginning of September, OIV Code N_o_ 304 – 3 -early), created by crossing the cultivars Queen of the Vineyard x Muscat Hamburg. It has a medium-sized cluster. The berries are medium-sized, oval with dark reddish blue skin. The flesh is medium firm with a delicate Muscat aroma.

Italia Muscat is a table cultivar of late ripening period (the beginning of October, OIV Code N_o_ 304 – 7 - late), created by crossing the cultivars Bicane x Muscat Hamburg. Its cluster is medium-sized or large, conical and loose. The berries are large, egg-shaped. The skin is thick, yellow-greenish or green-yellowish. The flesh is medium firm with a very pleasant muscat aroma.

Muscat Hamburg is a cultivar of late ripening period (the end of September, OIV Code N_o_ 304 - 7 - late). Its cluster is medium to large-sized, loose and branched. The skin is solid with dark blue color. The berries are oval, uneven in size with a fine Muscat aroma.

### 4.2. Chemical and Reagents

All chemicals of analytical reagent grade were obtained from Sigma-Aldrich (St. Louis, MO, USA).

### 4.3. Grape Brandy Making Technology

Grapes of each cultivar were harvested at the stage of its physiological full maturity, according to the method of O.I.V. [43]. For making the brandy, the technological process described in our previous paper [17], with some modifications, was applied. Prior to fermentation, and immediately after the disintegration of the grapes, a pectolytic enzyme was added in two different concentrations—0.3 g/kg and 0.7 g/kg of grape. The use of the pectolytic enzyme aimed to increase the intensity of the prefermentative aroma, due to its effect on the pomace and contribution to the hydrolysis of terpenic glycosides. In this study the enzyme “Gamapect LM”—Charge Q 586 (Gamma Chemie, Darmstadt—Germany) was used for this purpose.

Brandy samples obtained from each cultivar were labelled as: C—control (without the addition of the enzyme), V1 (0.3 g of pectolytic enzyme per kg of grape) and V2 (0.7 g of pectolytic enzyme per kg of grape). 

### 4.4. Extraction and Analysis of Volatile Compounds 

For all samples analyzed, liquid–liquid solvent extraction with pentane was applied (100 mL brandy and 10 mL pentane) for each sample. Analysis of volatile compounds was performed by gas chromatography-mass spectrometry (GC/MS) method as previously described [26]. Briefly, gas chromatographic analysis was performed using a gas chromatograph HP 5890 equipped with a flame ionization detector (FID) and a split/splitless injector. The separation was achieved using a HP—5 (5% diphenyl and 95% dimethylpolysiloxane) fused silica capillary column, 30 m × 0.25 mm i.d., 0.25 _m film thickness. GC oven temperature was programmed from 50 °C (6 min) to 285 °C at a rate of 4.3 °C/min. Hydrogen was used as carrier gas; flow rate was 1.6 mL/min at 45 °C. Injector temperature was 250 °C, detector temperature 280 °C, and injection mode splitless. An injection volume of 1.0 _L was used for the beverage extract. 

Gas chromatographic-mass spectrometric (GC/MS) analysis was performed using an Agilent 6890 gas chromatograph coupled with an Agilent 5973 Network mass selective detector (MSD), in positive ion electron impact (EI) mode. The separation was achieved using an Agilent 19091S-433 HP-5MS fused silica capillary column, 30 m × 0.25 mm i.d., 0.25 μm film thickness. GC oven temperature was programmed from 60 to 285 °C at a rate of 4.3 °C/min. Helium was used as carrier gas, inlet pressure was 25 kPa, linear velocity was 1 mL/min at 210 °C. Injector temperature was 250 °C, and injection mode splitless. MS scan conditions: source temperature, 200 °C; interface temperature, 250 °C; E energy, 70 eV; mass scan range, 40–350 amu (atomic mass units). Identification of compound was performed using both the retention index and comparison with reference spectra (Wiley database). The relative percentage of the compounds identified was computed from the GC peak area. 

### 4.5. Statistical Analysis

The principal component analysis (PCA) was used to study the similarity between Muscat cultivars, as well as the similarity of the measured parameters. This method is very useful when it is necessary to extract relevant information from complex datasets. This information was obtained by reducing the dimensionality of the space in which the measured data are scattered. The Principal components are linear combinations of the initial variables (oenological parameters) and thus the first main component is selected to absorb as much variance of the original data as possible, the second as much as possible from the remaining variance, etc. All Principal components were mutually orthogonal. The visualization of the PCA was achieved by the construction of the biplot, on which the cultivars were represented by points and the oenological parameters to the vectors. Agglomerative hierarchical clustering (AHC) was conducted in Addinsoft 2018.5 XLSTAT Sensory, MS Excel, Addinsoft NY USA. 

## 5. Conclusions

The results obtained in this study on volatile aromatic compounds in the analyzed grape brandies suggest significant differences in both the number of aromatic compounds and their relative content. Given the uniform grape brandy making technology, the differences observed were induced by the cultivars used for grape brandy production and possibly the length of their ripening period. The application of pectolytic enzyme did not influence evenly the increase of relative percentage of aromatic compounds in the examined brandy samples. Esters and terpenes were detected in the highest percentage, which may be attributed mainly to fruity and floral aromas of brandy samples. However, among terpenes, the main aroma contributors, the presence of limonene was associated only with the application of pectolytic enzyme. 

Principal Component Analysis revealed that esters and terpenes had the highest influence on discrimination between the Muscat cultivars. The Hierarchical Component Analysis of brandy samples without the addition of pectolytic enzyme (control) denotes a significant similarity among the cultivars, particularly between Muscat Hamburg and Italia Muscat, which may be attributed to the fact that Muscat Hamburg is one of the parents to the other examined cultivars. 

The initial examination of the applied enzyme effects imposes the need for further experiments and the use of other types of enzymes in order to compare their effectiveness in releasing aromatic compounds and improving brandy aroma.

## Figures and Tables

**Table 1:** Volatile aromatic compounds identified in Muscat table grape brandy.

Cultivar	Early Muscat (EM)			Radmilovac Muscat (RM)			Banat Muscat (BM)			Italia Muscat (IM)			Muscat Hamburg (MH)		
Treatments	C	V1	V2	C	V1	V2	C	V1	V2	C	V1	V2	C	V1	V2
							**ALCOHOLS**								
3-Ethoxy-1-propanol	/^2^	/	0.42^3^	0.72	/	/	/	/	/	/	/	/	0.26	/	1.71
4.4-Dimethyl-3-hexanol	1.51	/	/	/	/	/	/	/	/	/	/	/	/	/	/
1-Hexanol*	4.93	5.64		6.01	1.9	9.12	8.18	6.74	3.41		4.2	3.51	3.61	0.81	6.17
Phenyl–ethyl alcohol*	9.62	12.22	18.09	0.42	12.22	/	11.75	21.78	10.89	5.6	1.54	3.51	9.97	25.43	18.68
3-Methyl-1-pentanol	/	/	/	/	/	/	/	/	/	/	/	/	/	/	0.33
1-Heptanol	/	/	/	/	/	/	/	/	/	/	/	/	0.2	/	0.22
5-Hepten-2-ol	/	/	/	/	/	/	/	/	/	/	/	/	/	/	0.17
**∑**	16.06	30.08	18.51	6.43	14.15	9.12	19.93	28.52	14.3	5.6	5.74	7.02	14.04	26.24	27.28
							**ACIDS**								
Octanoic acid	0.32	0.63	0.88	/	/	/	/	0.1	1.25	5	2.59	/	0.11	/	1.95
Decanoic acid	1.25	4.91	6.01	/	1.58	0.27	2.91	/	/	/	0.5	8.41	/	/	/
Dodecanoic acid	5.2		6.61	/	1.01	1.8	/	/	2.19	2.23		3.83	0.11	3.18	1.24
Tetradecanoic acid	/	/	/	0.42	/	/	/	/	/	/	/	/	0.62	/	/
Hexadecanoic acid	0.51		0.68	3.11	3.41	0.62	/	/	/	/	/	/	0.33	/	/
Hexanoic acid	/	/	/	/	/	1.12	/	/	0.93	1.39	/	/	/	/	/
**∑**	7.28	5.54	14.18	3.53	6.0	3.81	2.91	0.1	4.37	8.62	3.09	12.24	1.17	3.18	3.19
							**ESTERS**								
Isoamyl acetate	/	/	0.17	/	/	/	/	/	/	/	/	/	/	/	/
Ethyl hexanoate*	4.96	0.84	1.91	1.01	0.39	2.04		1.97	0.58	1.71	9.8	7.19	4.58	4.97	4.32
Linalyl acetate	/	1.53	/	/	/	/	/	/	/	/	/	/	/	/	/
Ethyl benzoate	/	0.97	/	/	/	/	/	/	/	/	/	/	/	/	/
Ethyl octanoate*	2.82	/	2.78	4.35	5.89	8.53	12.33	15.17	10.68	16.96	13.04	27.56	24.83	/	31.45
2-Fenilethyl acetate	/	/	0.92	/	/	/	/	/	/	/	/	/	/	/	/
Citronellyl acetate	/	/	/	/	0.17	/	/	/	/	/	/	/	/	/	0.4
Neryl acetate	/	/	/	/	/	/	/	/	/	/	/	/	/	/	/
Ethyl-9-decanoate	/	/	/	/	/	/	/	/	/	1.09	/	/	/	/	/
Ethyl decanoate*	26.62	8.12	12.48	4.09	6.29	/	12.63	7.14	19.71	22.25	34.28	11.00	29.87	15.82	6.16
Isoamyl octanoate	/	/	/	/	/	/	/	/	/	/	/	/	0.27	/	0.11
Ethyl dodecanoate*	11.64	0.53	9.59	3.6	3.03	5.54	9.67	3.24	10.54	10.89	8.14	5.57	3.53	5.82	3.28
3-Methyl butyldecanoate	/	/	/	/	/	/	/	/	/	/	/	/	0.06	0.54	/
Ethyl tetradecanoate*	0.62	0.72	0.52	1.51	1.51	1.47	/	0.74	2.28	2.23	1.23	0.98	1.62	2.58	0.24
Ethyl 9-hexadecanoate*	0.51	0.32	/	1.35	0.75	/	/	2.18	4.48	1.14	1.55	0.46	0.23	1.75	0.24
Ethyl hexadecanoate*	1.54	3.65	3.88	6.73	10.78	14.43	15	11.39	14.26	12.33	6.98	7.49	2.3	10.57	2.12
Ethyl linoleate*	4.77	6.66	3.44	12.33	8.63	16.42	19.97	15.53	1.76	5.23	6.7	8.07	6.99	15.63	3.26
Ethyl stearate*	/	0.83	/	1.36	1.03	1.56	1.67	1.18	1.68	0.68	/	0.81	0.65	1.23	/
Diethyil-dibutanoate	/	/	/	/	0.21	/	/	/	0.25	/	/	/	0.28	/	0.54
Ethyl-3-hydroxy butyrate	/	/	/	/	/	/	/	/	/	/	/	/	0.19	/	/
3-Methylbutyl butanoate	/	/	/	/	/	/	/	/	/	/	/	/	/	0.46	/
**∑**	53.48	24.17	35.69	36.33	38.68	49.99	71.27	58.54	66.22	75.4	59.37	52.12	74.51	81.72	69.13
							**TERPENOIDS**								
α-Pinene	/	/	/	/	/	0.64	/	/	/	/	/	/	/	/	/
Limonene*	/	2.14	/	/	1.02	/	/	7.31	0.96	0.62	/	/	/	1.42	1.47
γ−Terpinene	/	/	/	/	0.79	/	/	/	/	/	/	/	/	0.86	0.59
*c*-Linalool oxide*	2.10	2.78	3.21	1.66	2.25	1.93	/	/	0.38	1.14	0.54	0.4	0.74	1.13	1.15
*t*-Linalool oxide*	0.56	0.61	3.37	0.23	2.30	0.39	/	/	/	0.47	0.3	/	0.41	0.92	0.73
α−Terpinolene	/	/	/	5.31	/	/	/	/	/	/	/	/	/	/	/
Linalool*	11.74	17.31	13.1	5.58	15.41	19.22	3.36	2.14	1.73	4.5	7.21	9.46	0.88	4.28	5.10
Hotrienol*	0.71	5.98	3.41	1.55	5.92	7.17	1.44	2.53	1.00	0.49	/	/	0.88	1.62	/
Rose oxide	/	/	/	/	0.28	/	/	/	/	/	/	/	/	/	/
Neroloxide		0.85	/	/	0.67	/	/	/	/	/	/	/	/	/	/
α-Terpineol	/	4.00	/	1.02	/	/	/	/	/	/	/	/	/	/	/
Citronellol*	/	17.05	2.46	2.63	4.76	4.72	/	0.49	5.50	1.72	/	1.21	4.75	0.3	7.95
Geraniol*	0.85	0.13	/	6.80	2.78	/	/	/	1.59	/	/	/	/	0.36	0.12
Farnesol	0.52	/	/	/	/	/	/	/	/	0.61	/	/	/	/	/
β-Fenchene	/	/	/	/	/	0.71	/	/	/	/	/	/	/	/	/
Epoxy-linalool	/	/	/	/	/	/	/	/	/	/	/	/	0.46	/	/
**∑**	16.48	50.85	25.55	24.78	36.18	34.78	4.8	12.47	11.16	8.12	10.89	17.11	9.55	8.05	11.07

C—control, V1—0.3 g of pectolytic enzyme per kg of grape; V2—0.7 g of pectolytic enzyme per kg of grape; /—not detected; Values for volatile aromatic compounds were given as relative abundance (%); *compounds used in PCA analysis

## Supplementary Materials

The following are available online, Table S1. Compounds identified in grape brandies produced from Muscat table grape cultivars and their aroma descriptors; Figure S1. Biplots of the volatile aroma compounds identified in grape brandy samples of each Muscat cultivar; Figure S2 Profile plot showing volatile composition of clusters 1–3 obtained by agglomerative hierarchal clustering analysis of samples without enzyme treatment (control); Figure S3 Profile plot showing volatile composition of clusters 1–3 obtained by agglomerative hierarchal clustering analysis of all samples with and without enzyme treatment; Figures S4-S18: Chromatograms of the volatile aromatic compounds in Early Muscat, Radmilovac Muscat, Banat Muscat, Italia Muscat and Muscat Hamburg grape brandy samples.
