# Supplementary material for: Volatile Aroma Compounds of Brandy ‘Lozovača′ Produced from Muscat Table Grapevine Cultivars (Vitis vinifera L.)
Source: Molecules. 2019 Jul 6;24(13):2485. doi: 10.3390/molecules24132485 (PMC6650811; doi:10.3390/molecules24132485)
Supplement: Supplementary file 1 [file molecules-24-02485-s001.pdf]

molecules

Supplementary Materials

Article

Volatile Aroma Compounds of Brandy 'Lozovača' Produced from Muscat Table Grapevine Cultivars (*Vitis vinifera* L.)

Saša Matijašević [1,*], Jelena Popović-Djordjević [2,*], Renata Ristić [3], Dušica Ćirković [4], Bratislav Ćirković [5] and Tatjana Popović [6]

[1] University of Belgrade, Faculty of Agriculture, Department of Horticulture, Nemanjina 6, 11080 Belgrade, Serbia
[2] University of Belgrade, Faculty of Agriculture, Department of Food Technology and Biochemistry, Nemanjina 6, 11080 Belgrade, Serbia
[3] University of Adelaide, ARC Training Centre for Innovative Wine Production, School of Agriculture, Food and Wine, PMB 1, Glen Osmond, SA 5064, Australia
[4] College of Agriculture and Food Technology, Ćirila i Metodija 1, 18400 Prokuplje, Serbia
[5] University of Priština, Faculty of Agriculture, Kopaonička bb, 38219 Lešak, Serbia
[6] University of Montenegro, Faculty of Biotechnology, Mihaila Lalića 1, 81000 Podgorica, Montenegro
* Correspondence: sasam@agrif.bg.ac.rs (S.M.); jelenadj@agrif.bg.ac.rs (J.P.-D.); Tel.: +38-111-441-3351 (S.M.); +38-111-441-3142 (J.P-D.)

molecules

MDPI

Supplementary Tables

Table S1. Compounds identified in grape brandies produced from Muscat table grape cultivars and their aroma descriptors

Class	Compound	Aroma descriptor
Alcohol	1-Hexanol	Flower [27, 29, 40ʹ], green [27, 29, 40], cut grass [29, 40], grass [27], herbaceous [23, 27]ʹ
	Phenyl-ethyl alcohol	Floral [27, 40], rose [27,40], honey [27]
	1-Heptanol	Oily [40]
Acid	Octanoic acid	Rancid [23], cheese [23, 40] fatty [40], sweet [40]
	Hexadecanoic acid	Rancid [23], cheese [25], fatty [25], sweet [40]
Ester	Ethyl hexanoate	Fruity[23,25], green apple [25, 29, 40], banana [23,25], wine-like [23,25], brandy [25]
	Ethyl octanoate	Sweet [23, 40], floral [23], fruity [23, 40], banana [23], pear]23], brandy [23]
	Ethyl decanoate	Fruity, brandy, grape [37]
Terpenoid	α-Pinene	Pine, resinous [31]
	Limonene	Fruity, lemon [41]
	γ-terpinene	Fruity, lemon-like [31]
	c-Linalool oxide	Floral [40]
	t-Linalool oxide	Woody, floral [40]
	Rose oxide	Floral [24]ʹ, lychee-like [24], rose [37]
	Linalool	Roses, anise seed, grapefruit, green lemon and citrus [15,41]
	Hotrienol	Fresh, floral, fruity [15,41]
	Citronellol	Roses, anise seed, grapefruit, green lemon and citrus [15,41]

Supplementary Figures

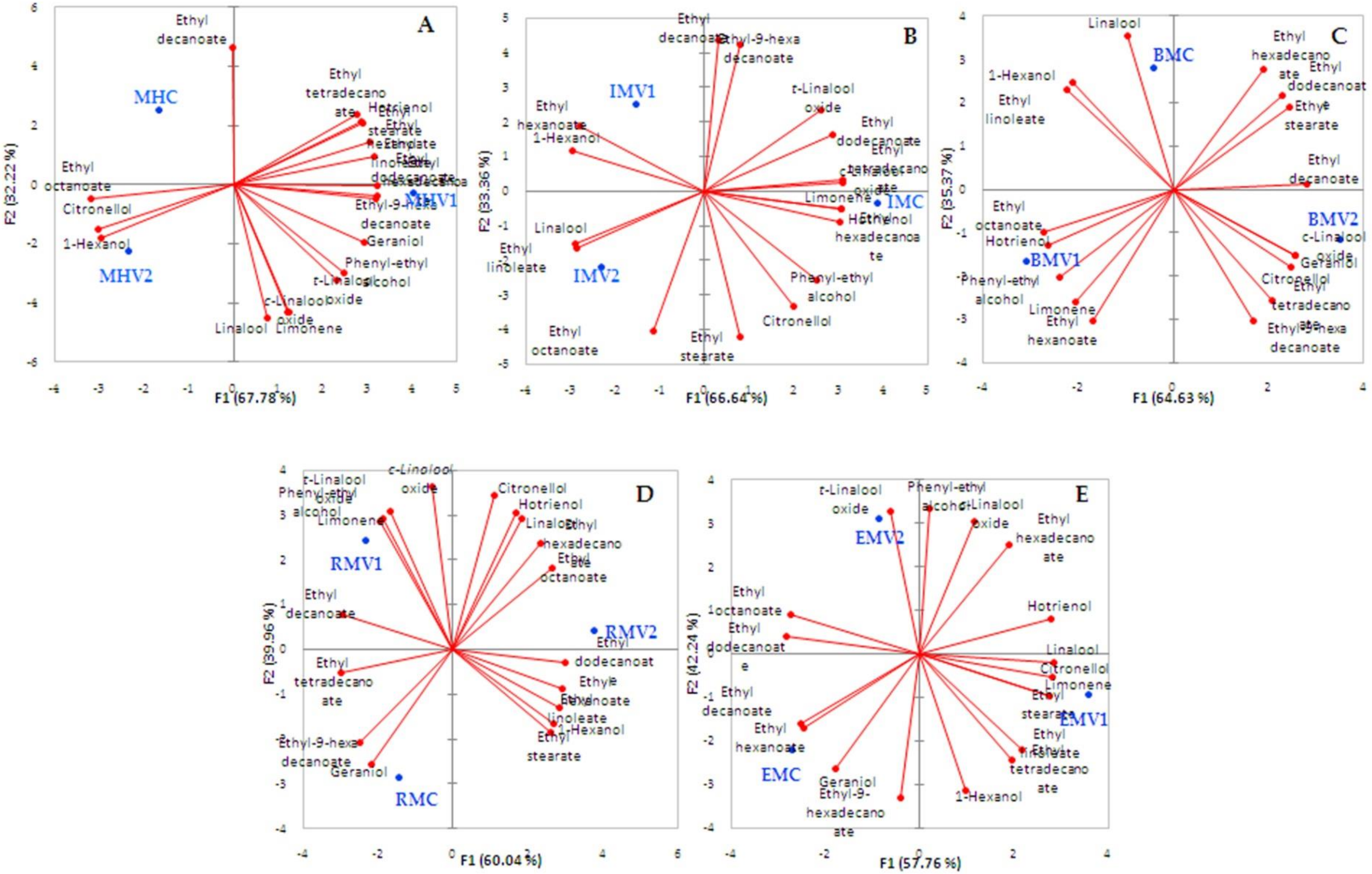

Figure S1. Biplots of the volatile aroma compounds identified in grape brandy samples of each Muscat cultivar.

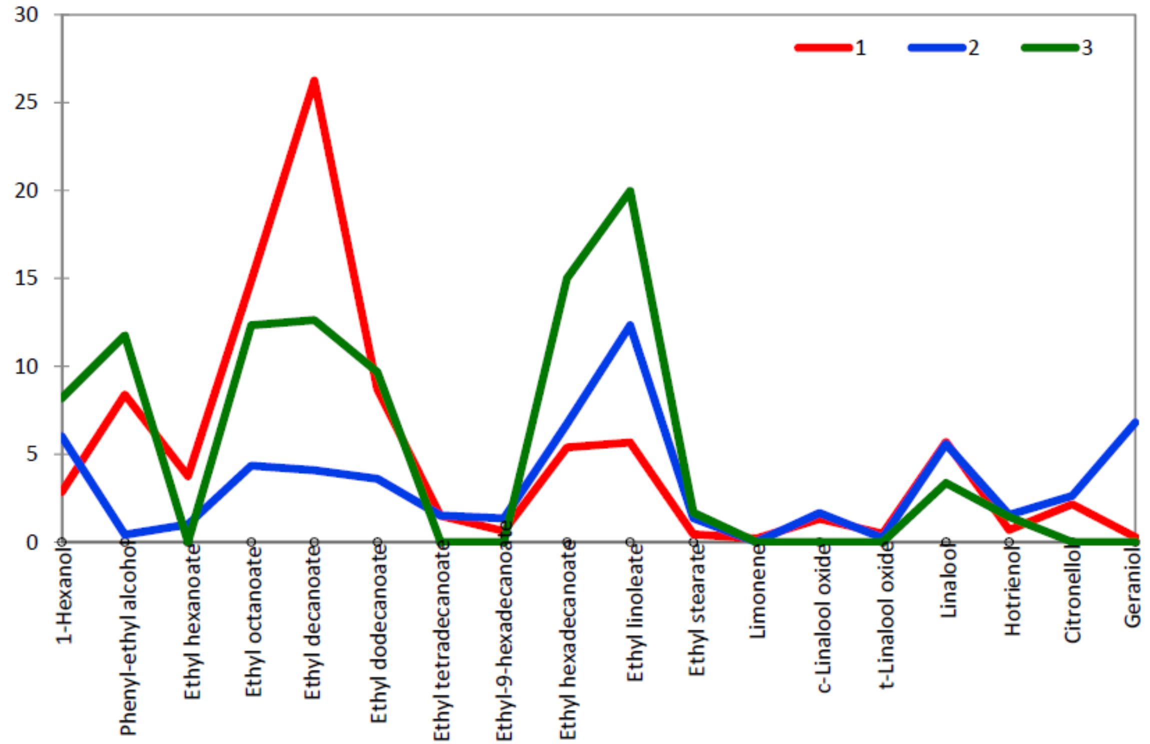

Figure S2. Profile plot showing volatile composition of clusters 1-3 obtained by agglomerative hierarchal clustering analysis of samples without enzyme treatment (control)

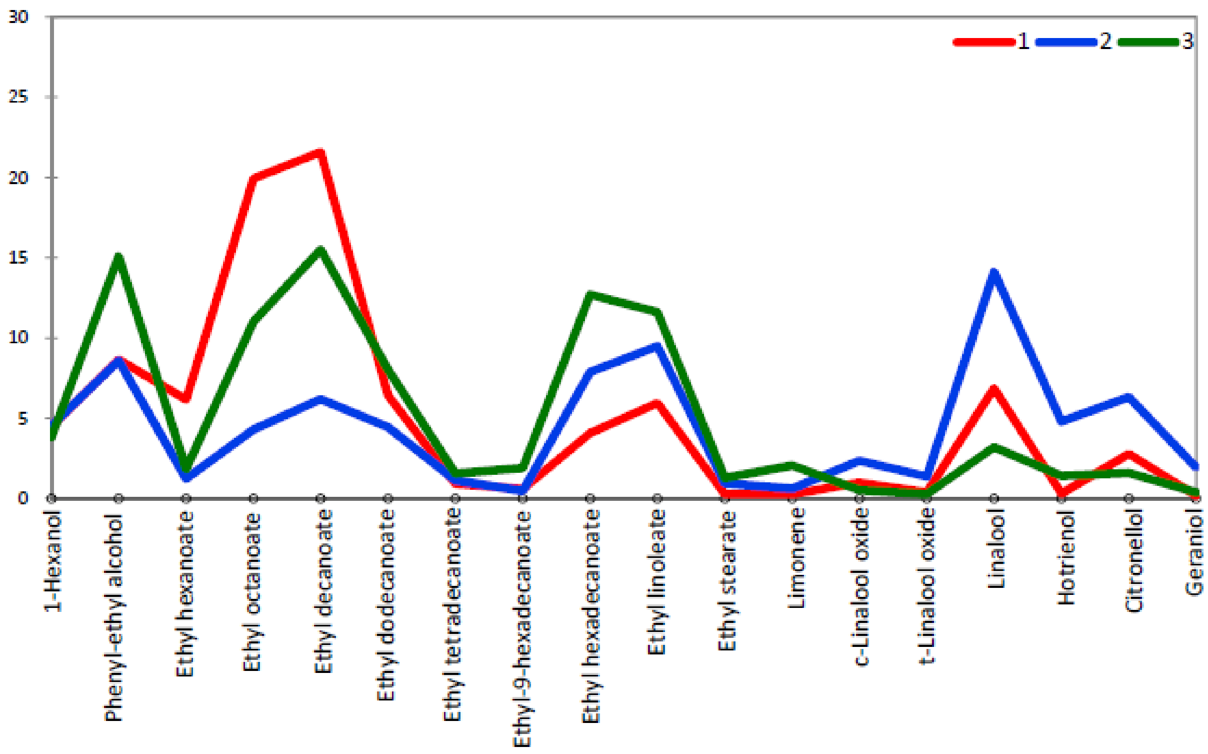

Figure S3. Profile plot showing volatile composition of clusters 1-3 obtained by agglomerative hierarchal clustering analysis of all samples with and without enzyme treatment

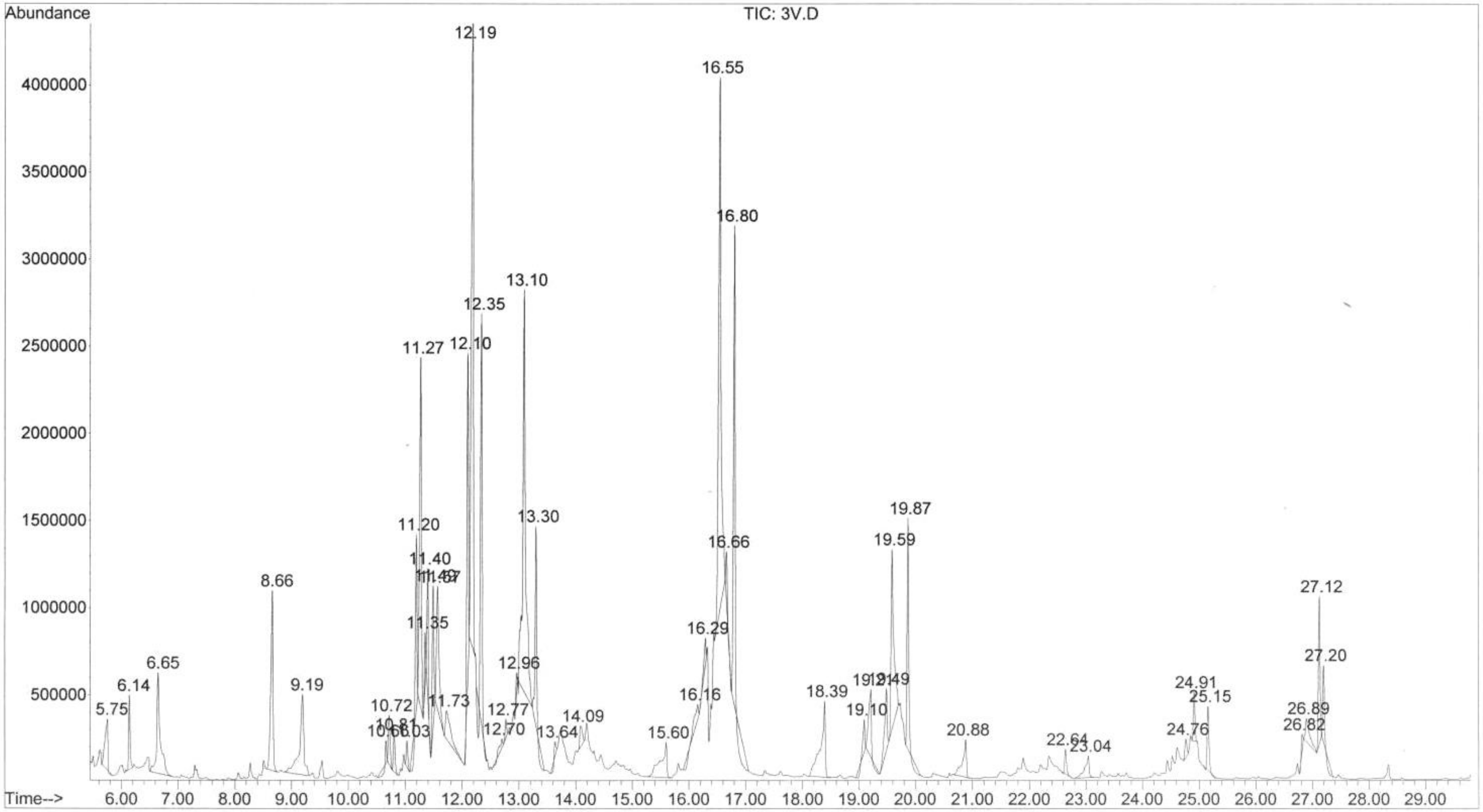

Figure S4. Chromatogram of the volatile aromatic compounds in Early Muscat grape brandy control - EMC

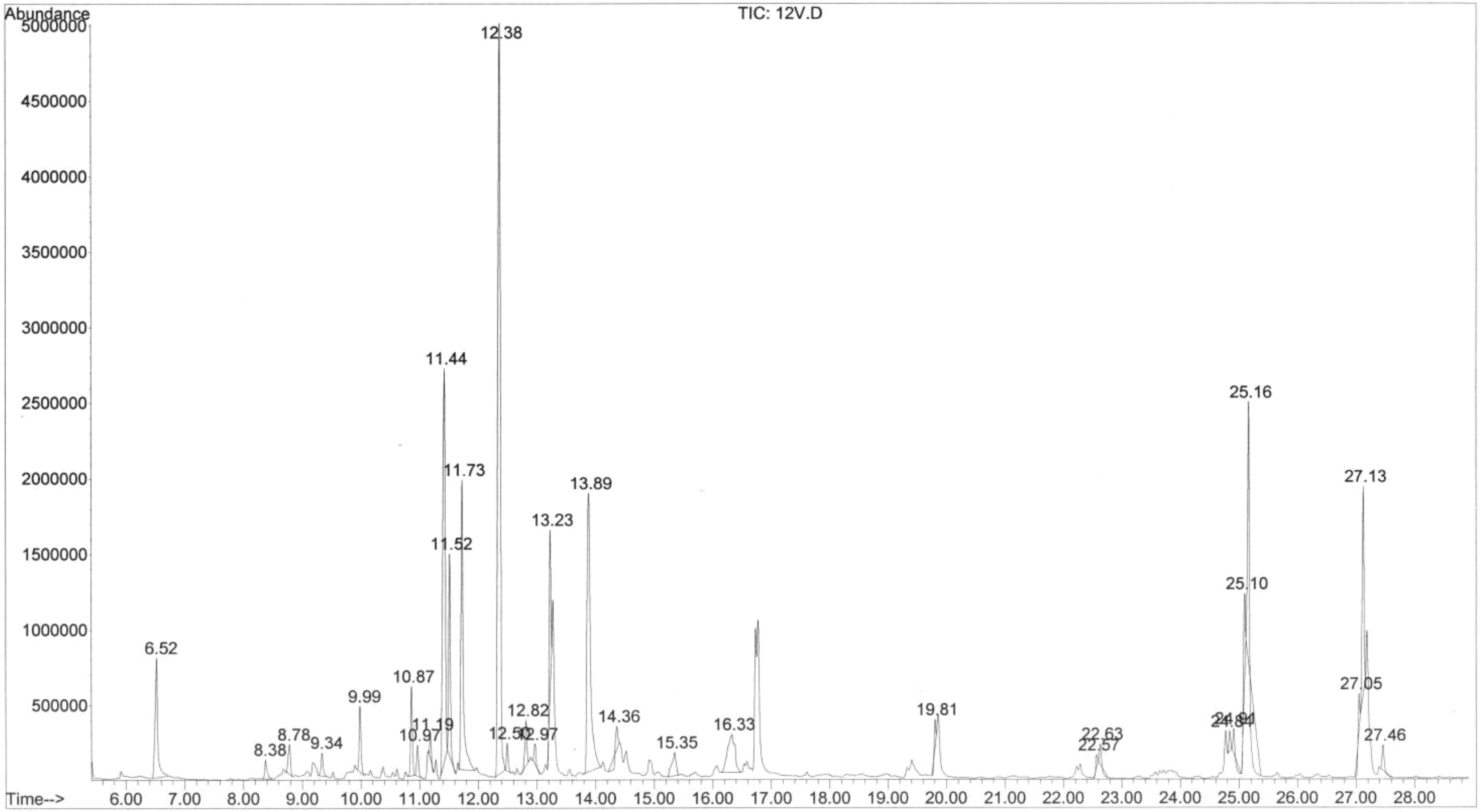

Figure S5. Chromatogram of volatile aromatic compounds in Early Muscat grape brandy with the addition of pectolytic enzyme - EMV1

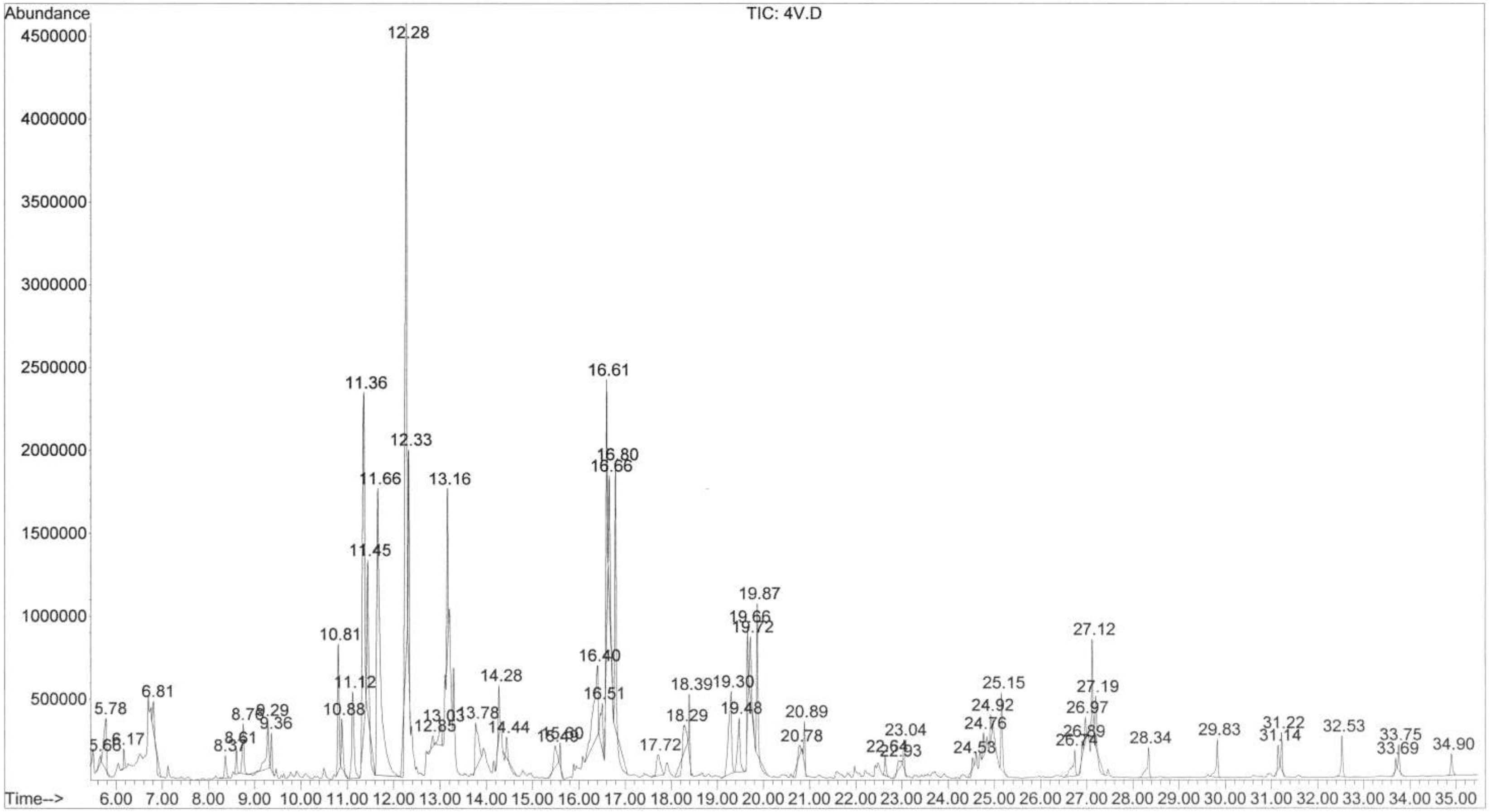

Figure S6. Chromatogram of volatile aromatic compounds in Early Muscat grape brandy with the addition of pectolytic enzyme – EMV2

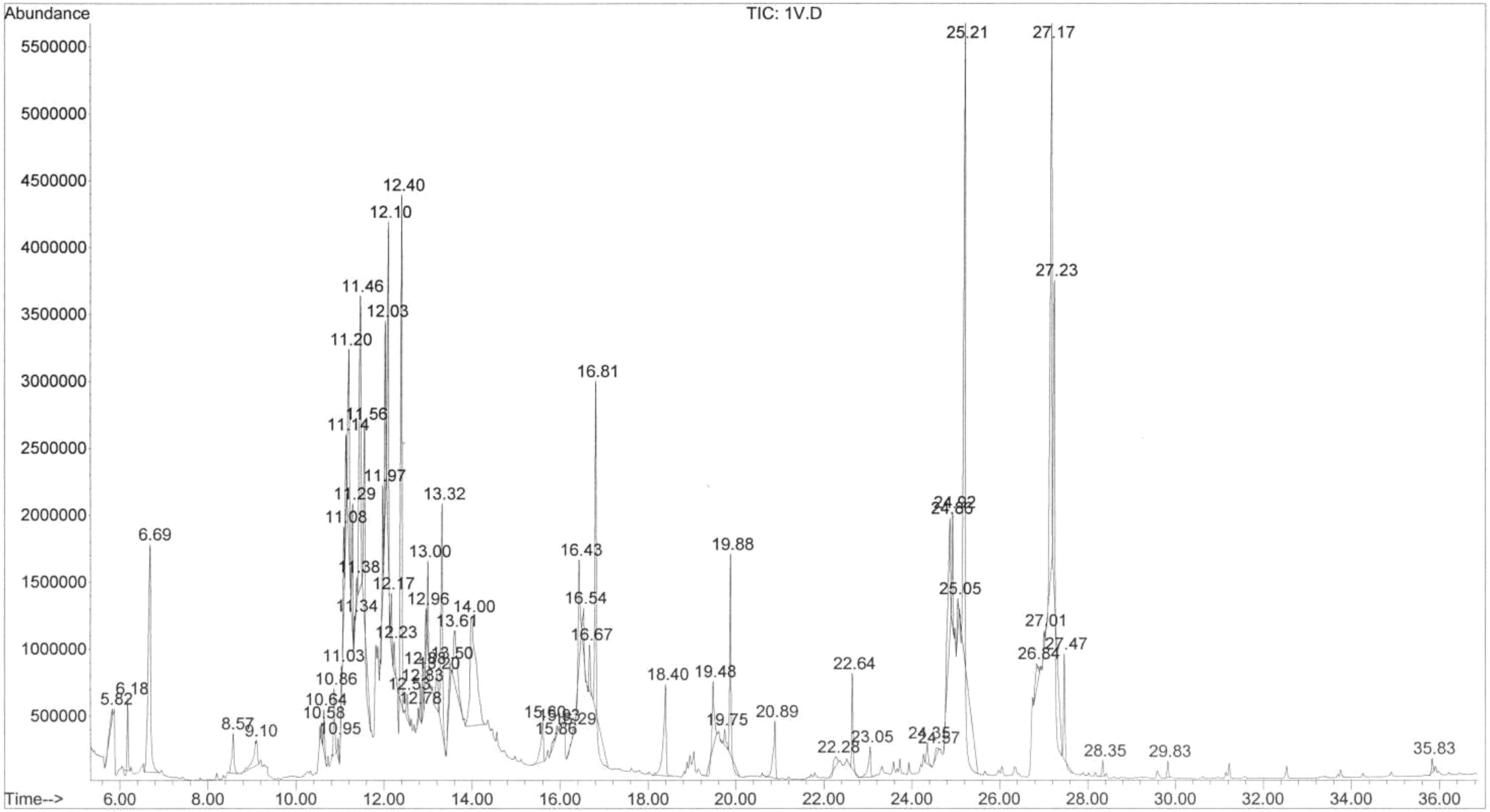

Figure S7. Chromatogram of volatile aromatic compounds in Radmilovac Muscat grape brandy control - RMC

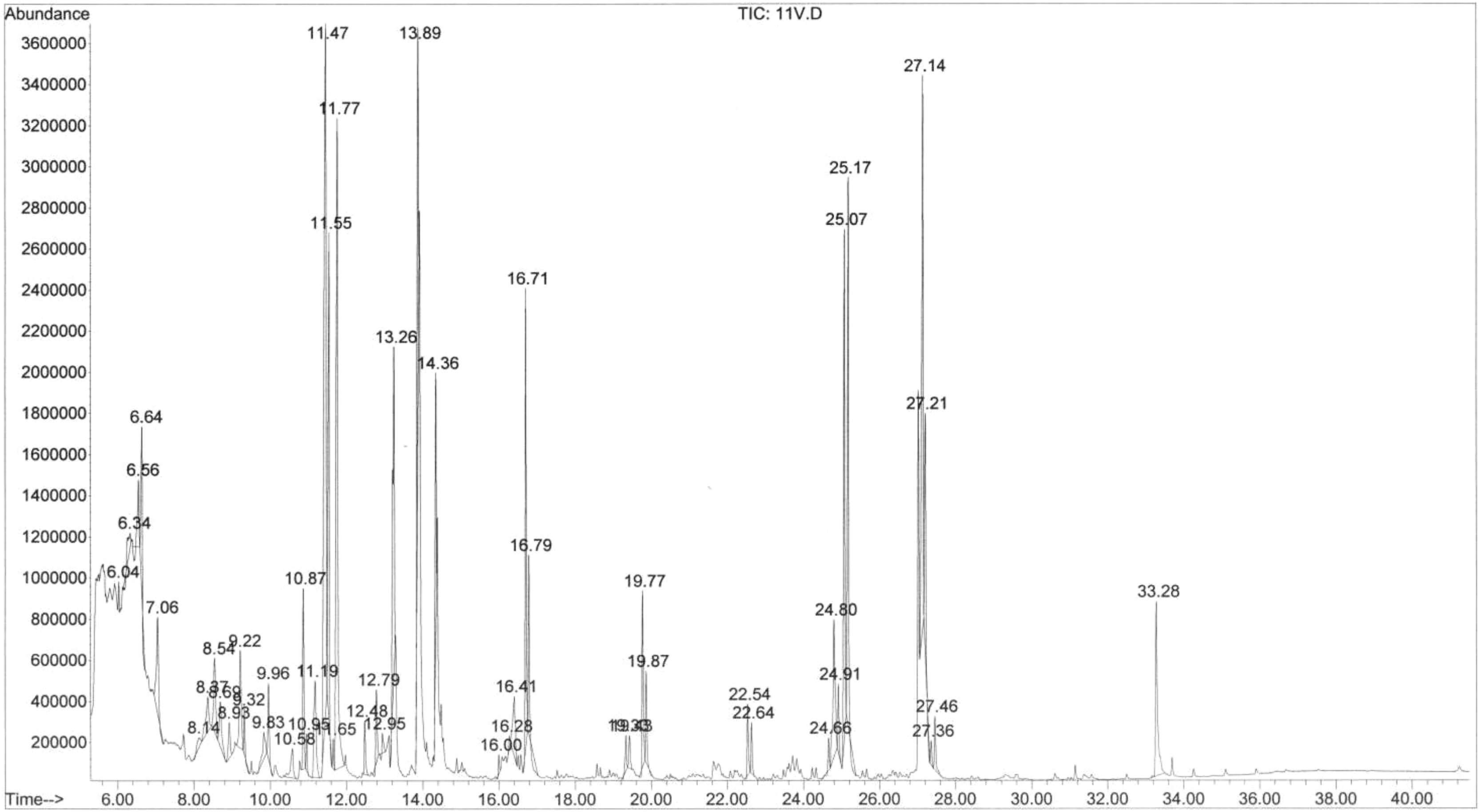

Figure S8. Chromatogram of volatile aromatic compounds in Radmilovac Muscat grape brandy with the addition of pectolytic enzyme - RMV1

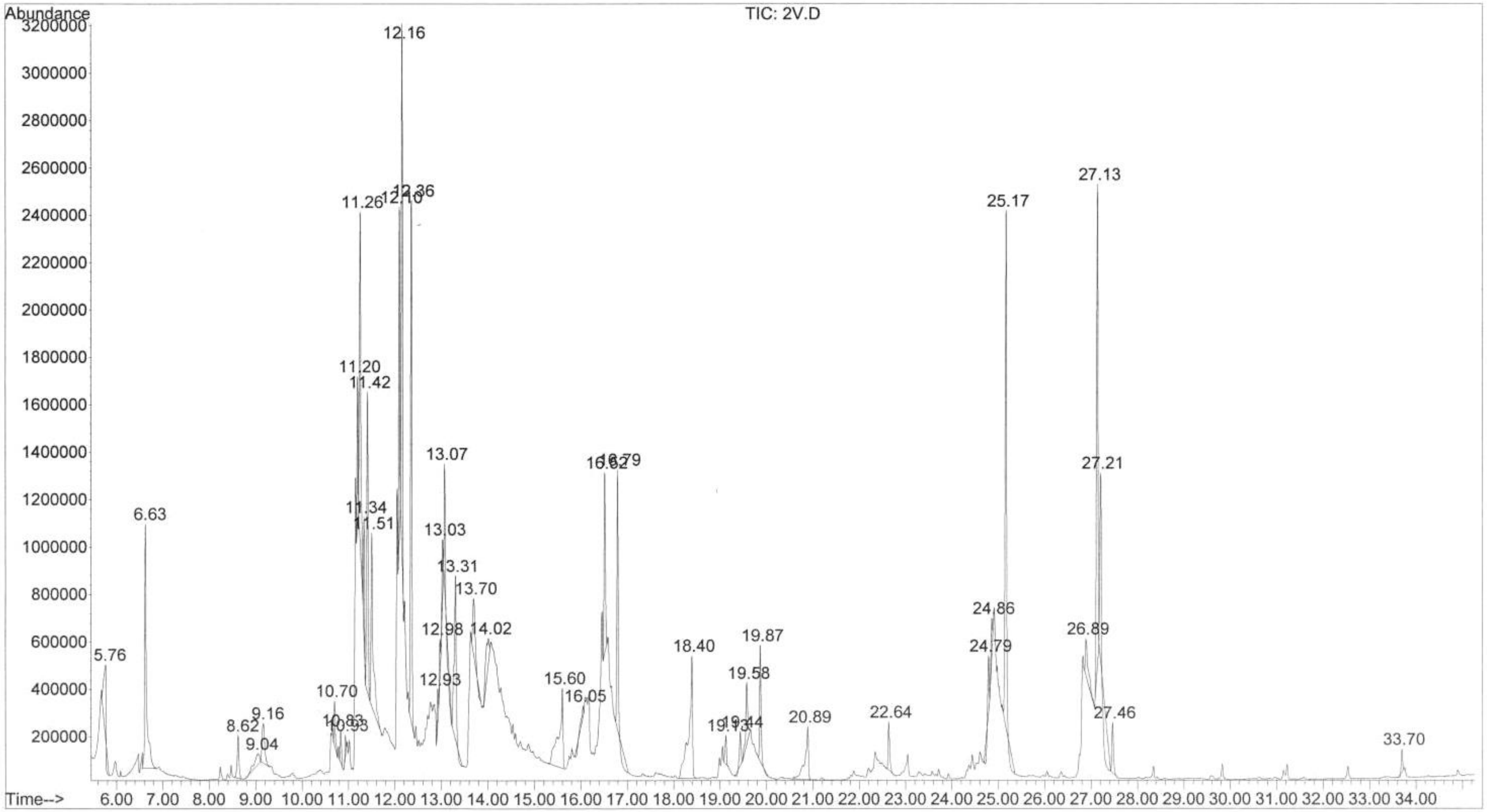

Figure S9. Chromatogram of volatile aromatic compounds in Radmilovac Muscat grape brandy with the addition of pectolytic enzyme – RMV2

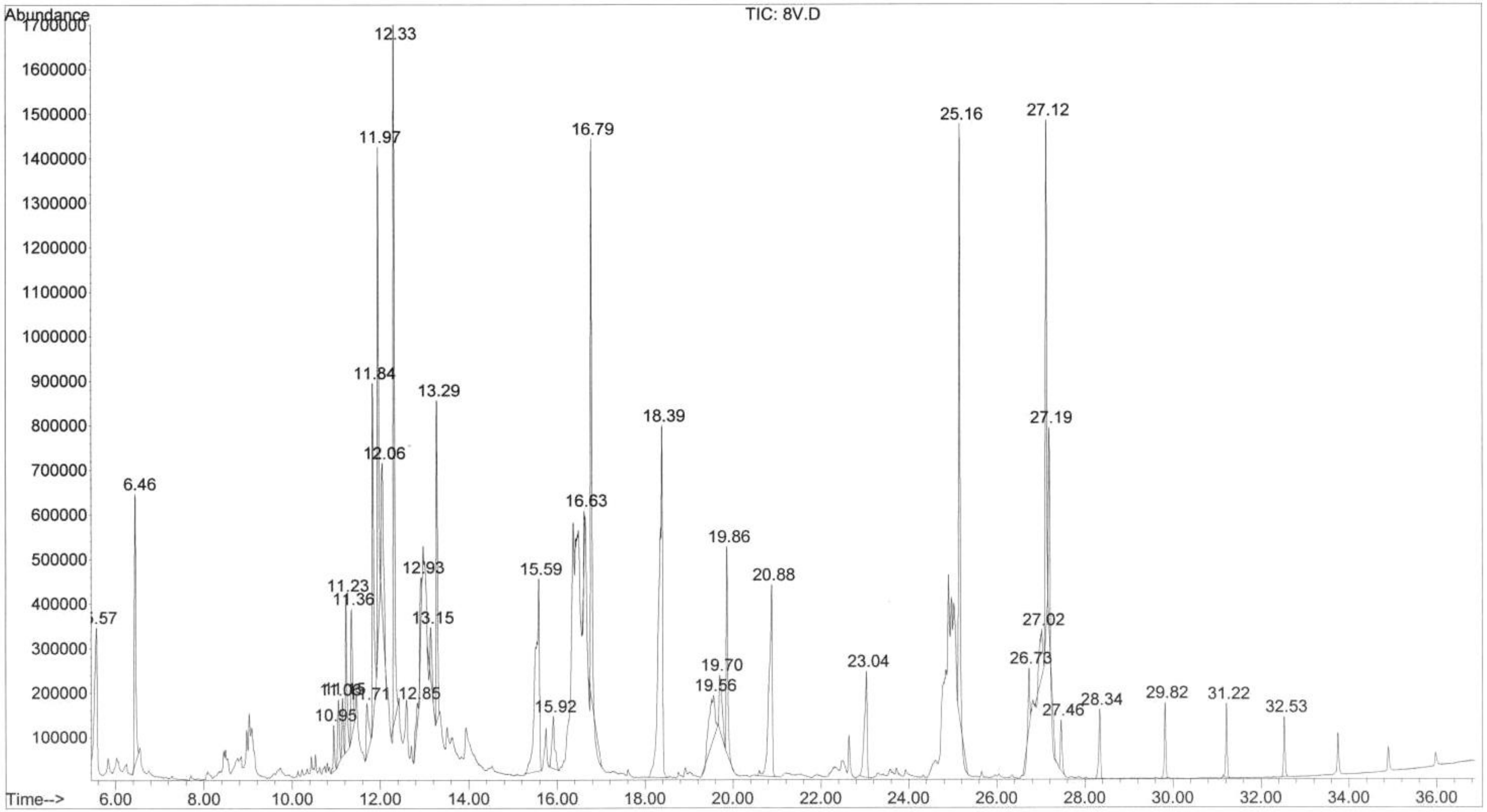

Figure S10. Chromatogram of volatile aromatic compounds in Banat Muscat grape brandy control - BMC

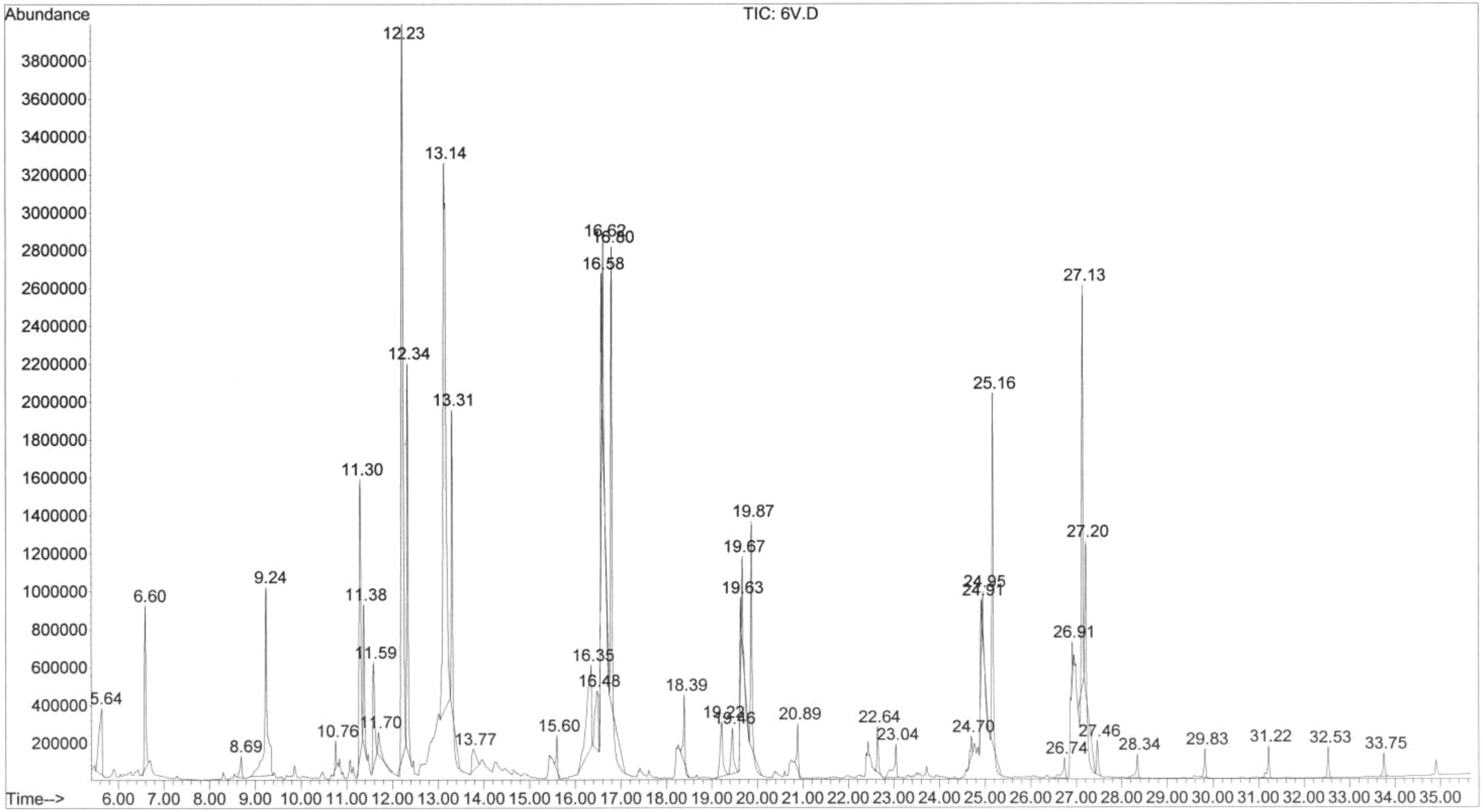

Figure S11. Chromatogram of the volatile aromatic compounds in Banat Muscat grape brandy with the addition of pectolytic enzyme - BMV1

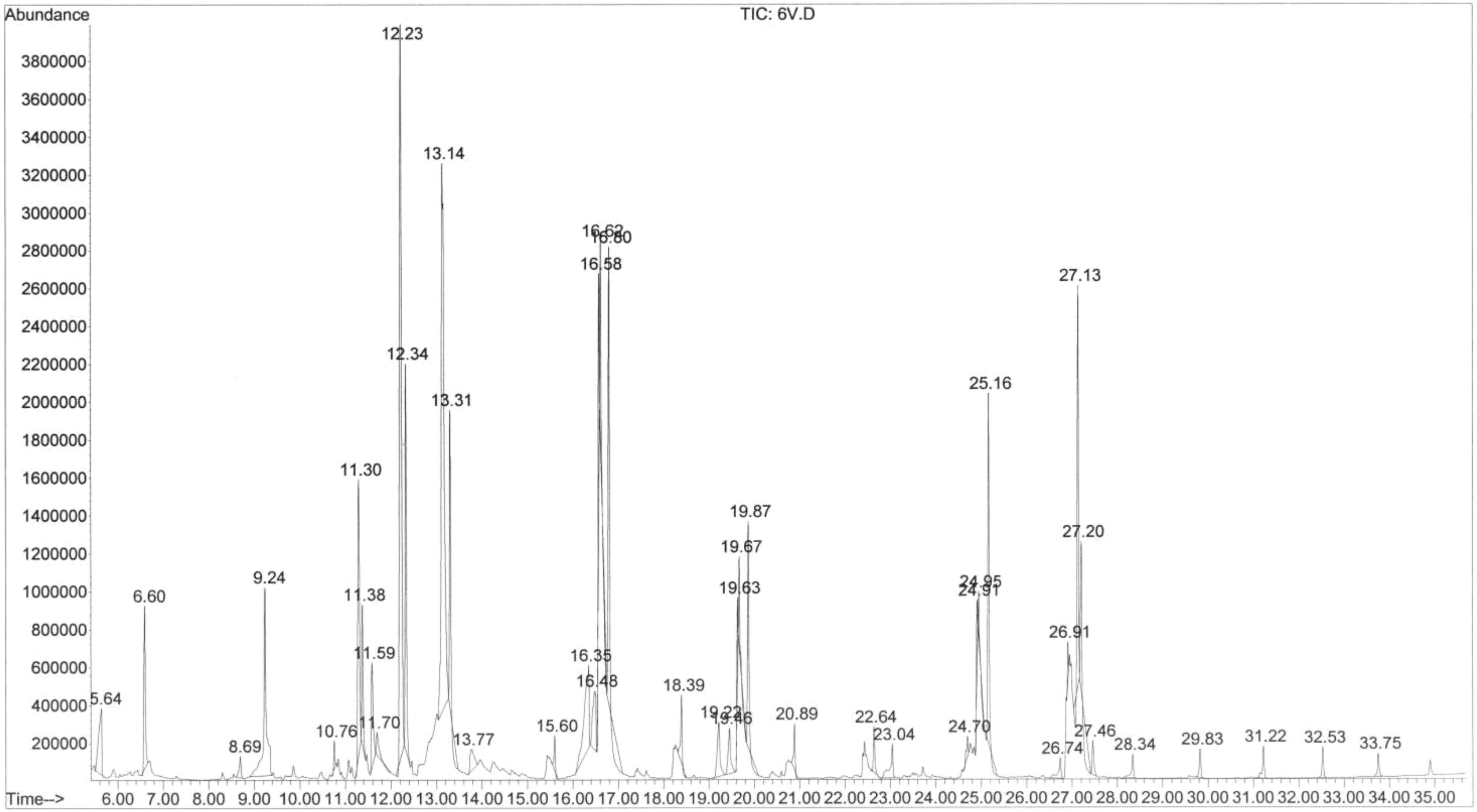

Figure S12. Chromatogram of volatile aromatic compounds in Banat Muscat grape brandy with the addition of pectolytic enzyme – BMV2

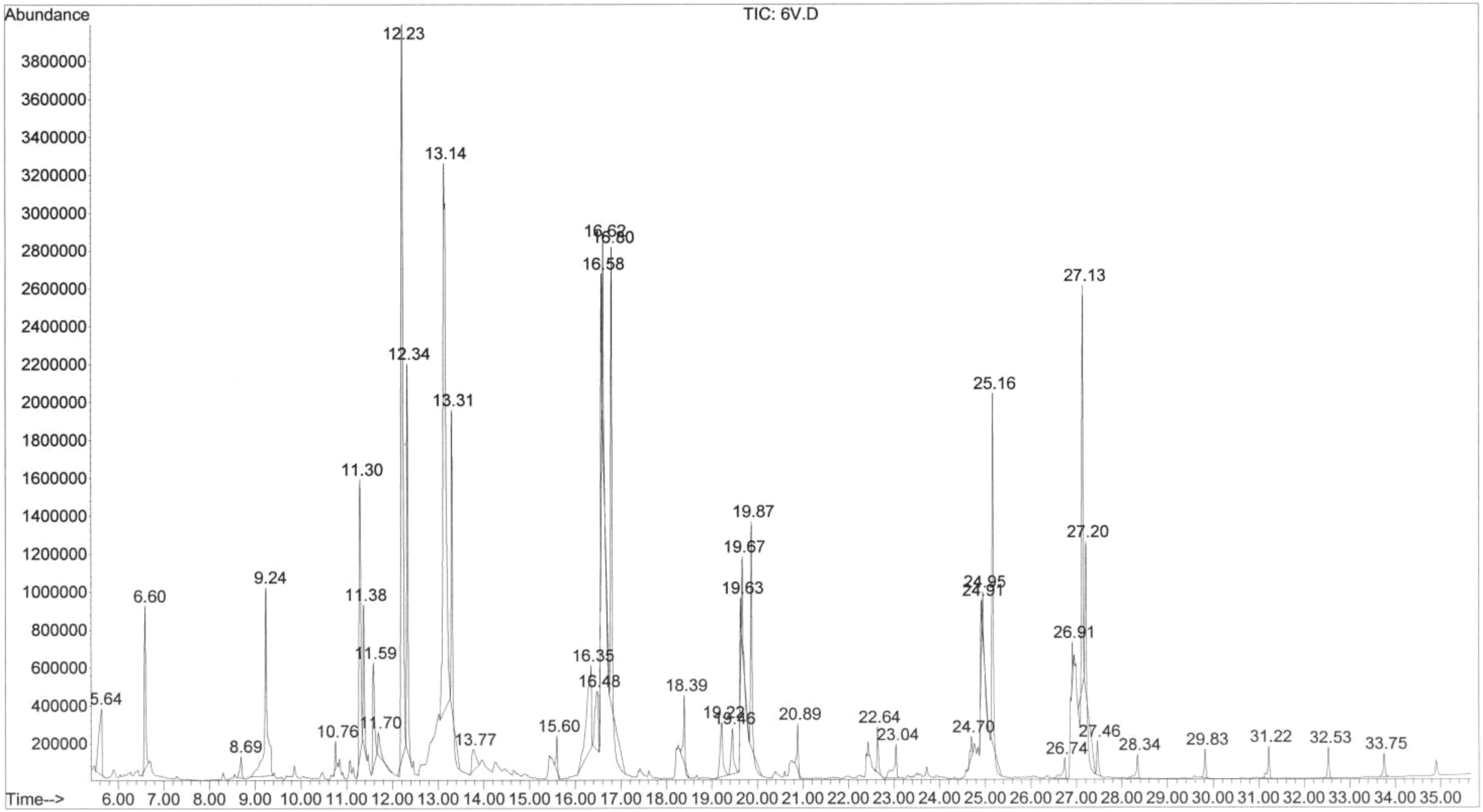

Figure S13. Chromatogram of volatile aromatic compounds in Italia Muscat grape brandy control - IMC

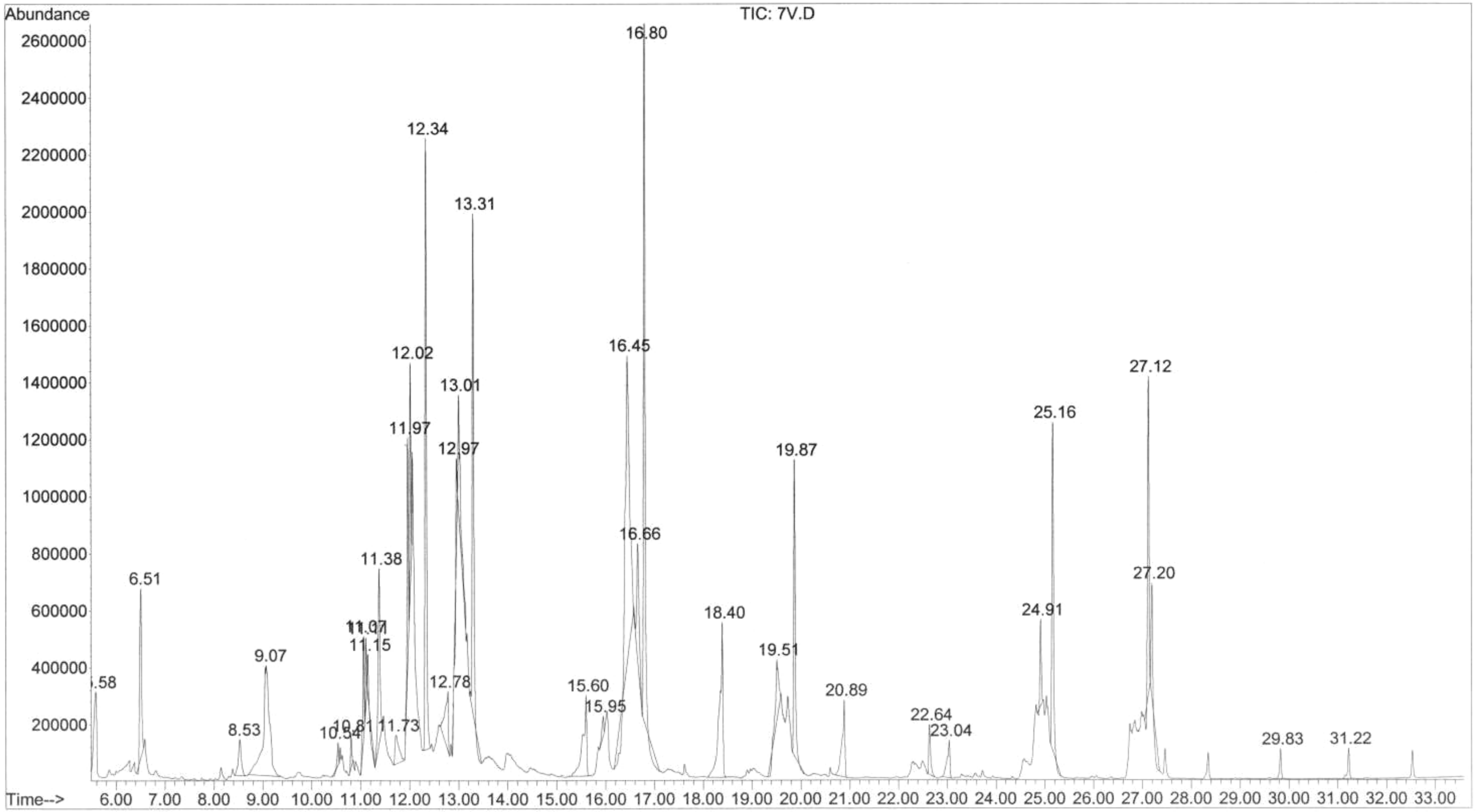

Figure S14. Chromatogram of volatile aromatic compounds in Italia Muscat grape brandy with the addition of pectolytic enzyme **–** IMV1

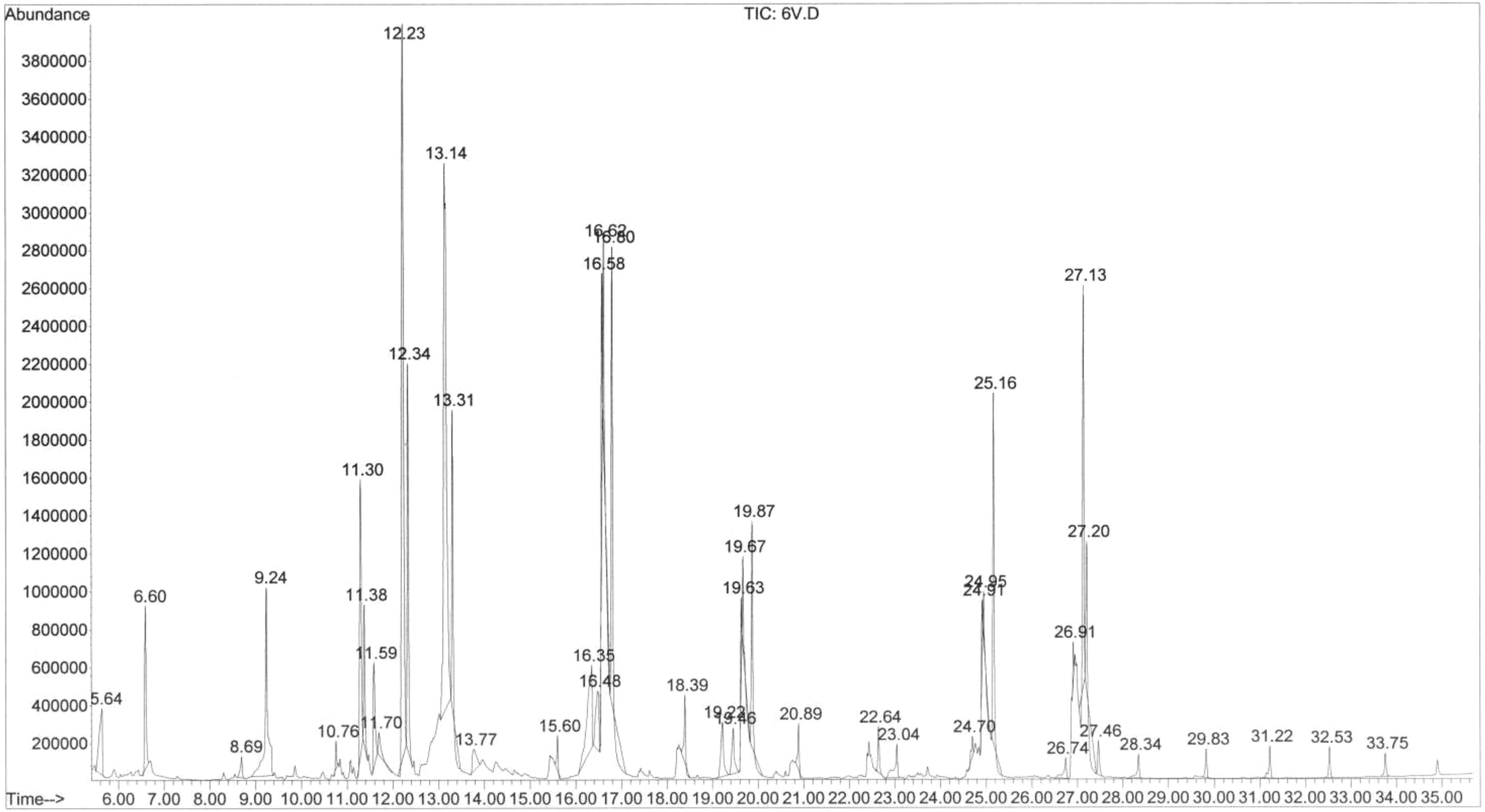

Figure S15. Chromatogram of volatile aromatic compounds in Italia Muscat grape brandy with the addition of pectolytic enzyme – IMV2

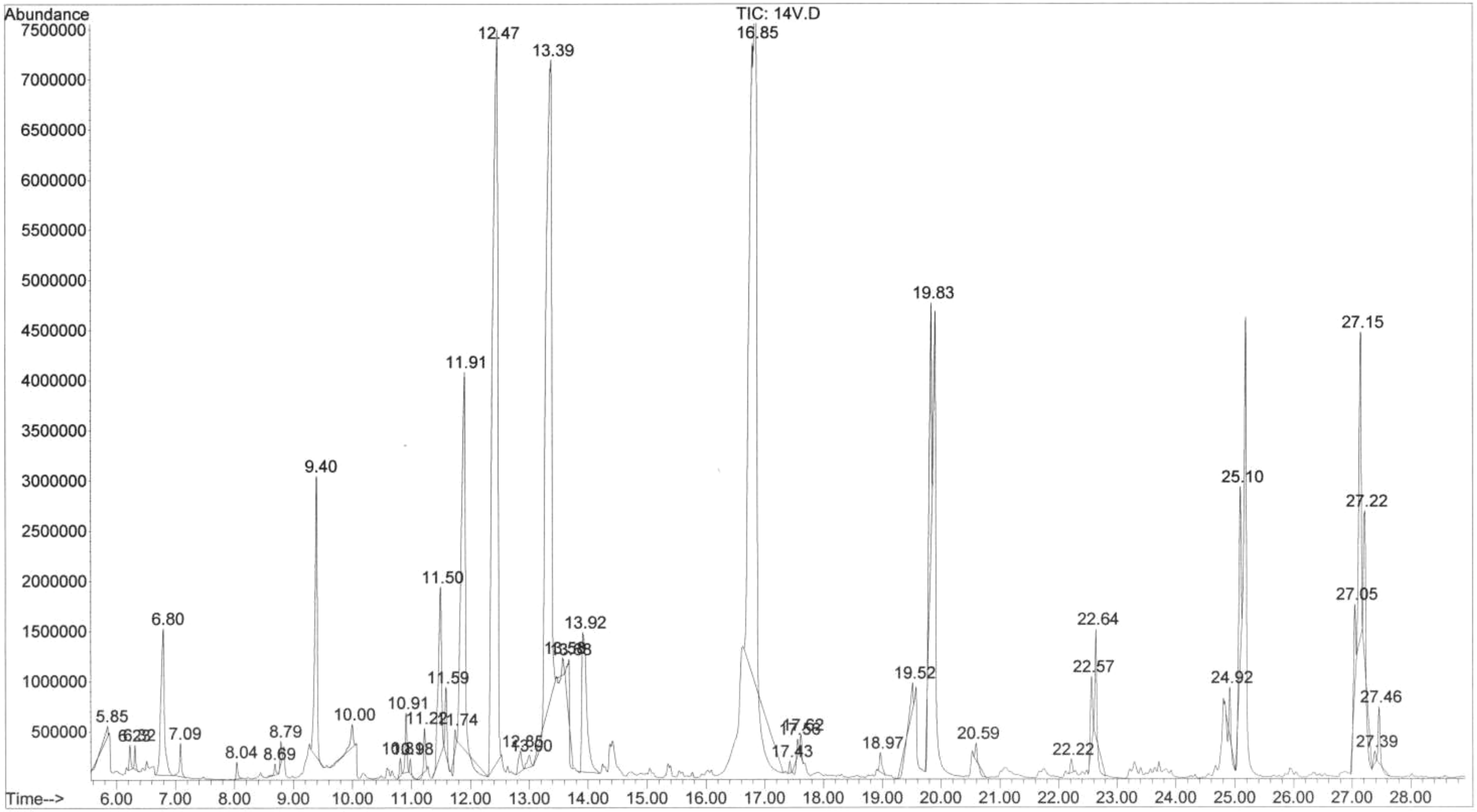

Figure S16. Chromatogram of volatile aromatic compounds in Muscat Hamburg grape brandy control – MHC

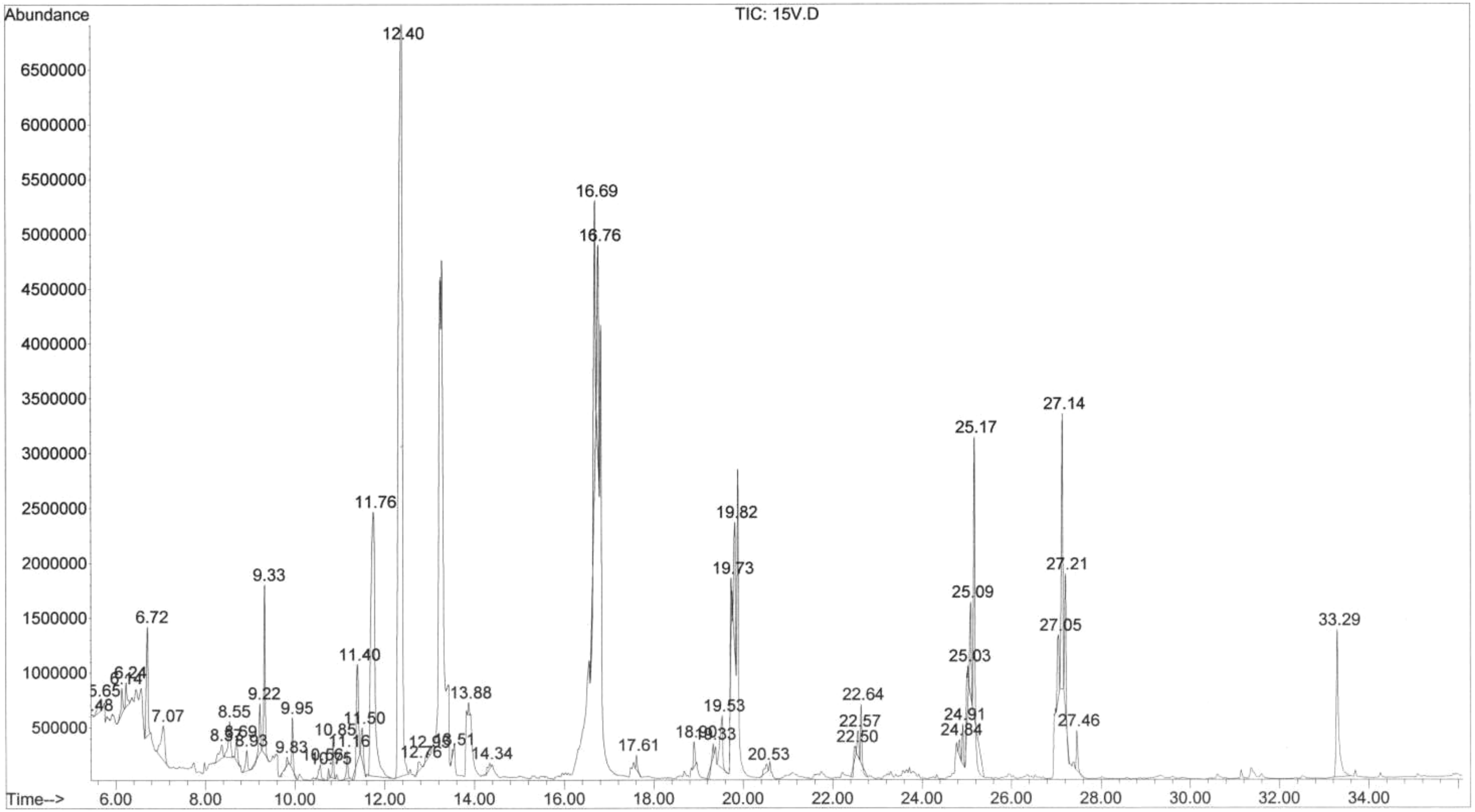

Figure S17. Chromatogram of volatile aromatic compounds in Muscat Hamburg grape brandy with the addition of pectolytic enzyme – MHV1

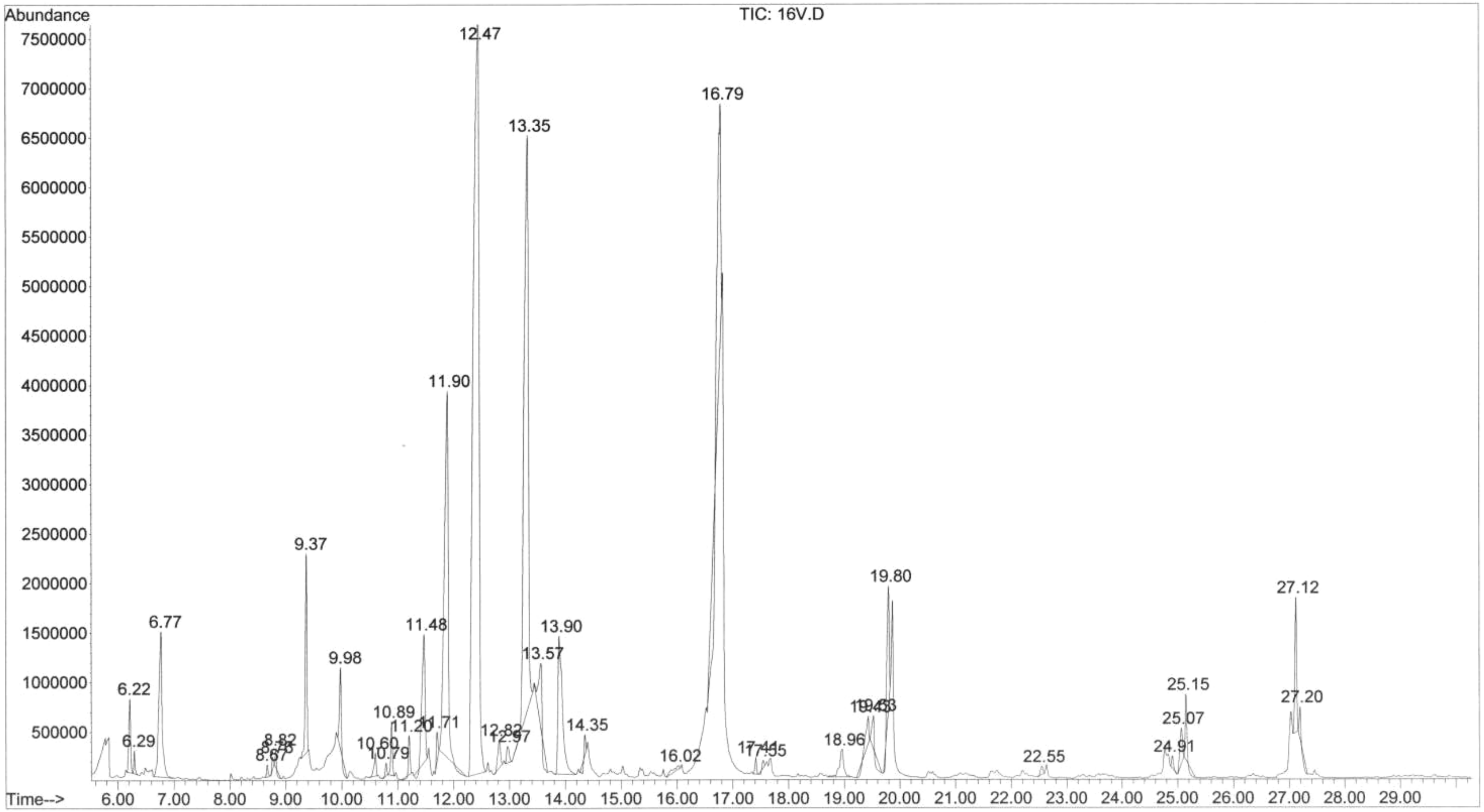

Figure S18. Chromatogram of volatile aromatic compounds in Muscat Hamburg grape brandy with the addition of pectolytic enzyme – MHV2

References

23. Peinado, R.A.; Mauricio, J.C.; Moreno, J. Aromatic series in sherry wines with gluconic acid subjected to different biological aging conditions by *Saccharomyces cerevisiae* var. capensis. *Food Chem.* **2006,** *94*, 232–239.
24. Mahattanatawee, K.; Perez-Cacho, P.R.; Davenport, T.; Rouseff, R. Comparison of three lychee cultivar odor profiles using gas chromatography-olfactometry and gas chromatography-sulfur detection. *J. Agric. Food Chem.* **2007**, *55*, 1939–1944.
25. Peinado, R.A.; Moreno, J.; Bueno, J.E.; Moreno, J.A.; Mauricio, J.C.; Comparative study of aromatic compounds in two young white wines subjected to pre-fermentative cryomaceration. *Food Chem.* **2004**, *84,* 585–590.
27. Genovese, A.; Lamorte, S.A.; Gambuti, A.; Moio, L. Aroma of Aglianico and Uva di Troia grapes by aromatic series. *Food Res. Int.* **2013,** *53*, 15–23.
29. Garcia-Carpintero, E.G.; Sanchez-Palomo, E.; Gomez Gallego, M.A.;Gonzalez-Vinas, M.A. Effect of cofermentation of grape varieties on aroma profiles of La Mancha red wines. *J. Food Sci.* **2011**, *76*, C1169–C1180
31. Qian, M.C.; Wang, Y. Seasonal variation of volatile composition and odor activity value of 'Marion' (*Rubus spp. hyb*) and 'Thornless Evergreen' (*R. laciniatus* L.) blackberries. *J. Food Sci.* **2005**, *70*, C13–C20.
37. Wu, Y.; Zhu, B.; Tu, C.; Duan, C.; Pan, Q. Generation of volatile compounds in litchi wine during winemaking and short-term bottle storage. *J. Agric. Food Chem*. **2011**, *59*, 4923–4931.
40. García-Carpintero, E.G.; Sánchez-Palomo, E.; Gallego, M.A.G.; González-Viñas, M.A. Volatile and sensory characterization of red wines from cv. Moravia Agria minority grape variety cultivated in La Mancha region over five consecutive vintages. *Food Res. Int.* **2011,** *44*, 1549–1560.
41. Diéguez, C.S.; de la Peňa, M.L.G.; Gómez F.E. Approaches to spirit aroma: contribution of some aromatic compounds to the primary aroma in samples of Orujo spirits. *J. Agr. Food Chem*. **2003**, *51*, 7385–7390. doi: 10.1021/jf0302916
